# Supplementary material for: The cell cycle and cell size influence the rates of global cellular translation and transcription in fission yeast
Source: EMBO J. 2023 Mar 23;42(9):e113333. doi: 10.15252/embj.2022113333 (PMC10152140; doi:10.15252/embj.2022113333)
Supplement: Supplementary file 1 — Expanded View Figures PDF [file EMBJ-42-e113333-s002.pdf]

Expanded View Figures

Figure EV1. The assays reliably measure RNA and protein production.

A Same as Fig 1B with time 0 included. The scale bar represents 5 μm.
B Growth curves of PN1 in EMM with or without 10 μM HPG at 25°C as measured by the change in optical density at 595 nm (OD595) over time. The doubling time of each condition indicated in the legend is calculated using the slope of the OLS linear regression fitted on the data after a natural logarithmic transformation.
C HPG signal shown in Fig 1C before normalisation and background removal.
D Wild-type cells (PN1) were incubated for 30 min with 10 μM HPG, then assayed for global translation, treated with 0.05 mg/ml proteinase K at 55°C for 4 h and fluorescence was measured using flow cytometry. The 0.05, 0.25, 0.5, 0.75 and 0.95 population quantiles of at least 200,000 cells are shown.
E Wild-type cells (PN1) were spun down and resuspended in EMM (control) or EMM + 10 mg/ml cycloheximide (t = 0), then assayed for global translation at different times using flow cytometry. Population medians of at least 200,000 cells are shown.
F Cells expressing *hENT1* (PN6002), *hsvTK* (PN6003) or both (PN10597) were pulsed with 10 μM EU for 30 min and assayed for global transcription using flow cytometry. The 0.05, 0.25, 0.5, 0.75 and 0.95 population quantiles of at least 200,000 cells are shown.
G Same as Fig 1E with time 0 included. The scale bar represents 5 μm.
H Same as (B) for PN10597 grown with or without 20 μM EU.
I EU signal shown in Fig 1F before normalisation and background removal.
J Cells expressing *hENT1* and *hsvTK* (PN10597) were pulsed with 10 μM EU and labelled with Alexa Fluor 488 azide, then treated with 0.1 mg/ml RNase A at 37°C for 16 h, and the fluorescence signal was assessed using flow cytometry. The 0.05, 0.25, 0.5, 0.75 and 0.95 population quantiles of at least 200,000 cells are shown.
K Cells expressing *hENT1* and *hsvTK* (PN10597) were pulsed with EU plus DMSO or EU plus 300 μg/ml 1,10-phenanthroline, and global transcription was assayed at different time intervals using flow cytometry. Population medians of at least 200,000 cells are shown.

© 2023 The Francis Crick Institute

© 2023 The Francis Crick Institute

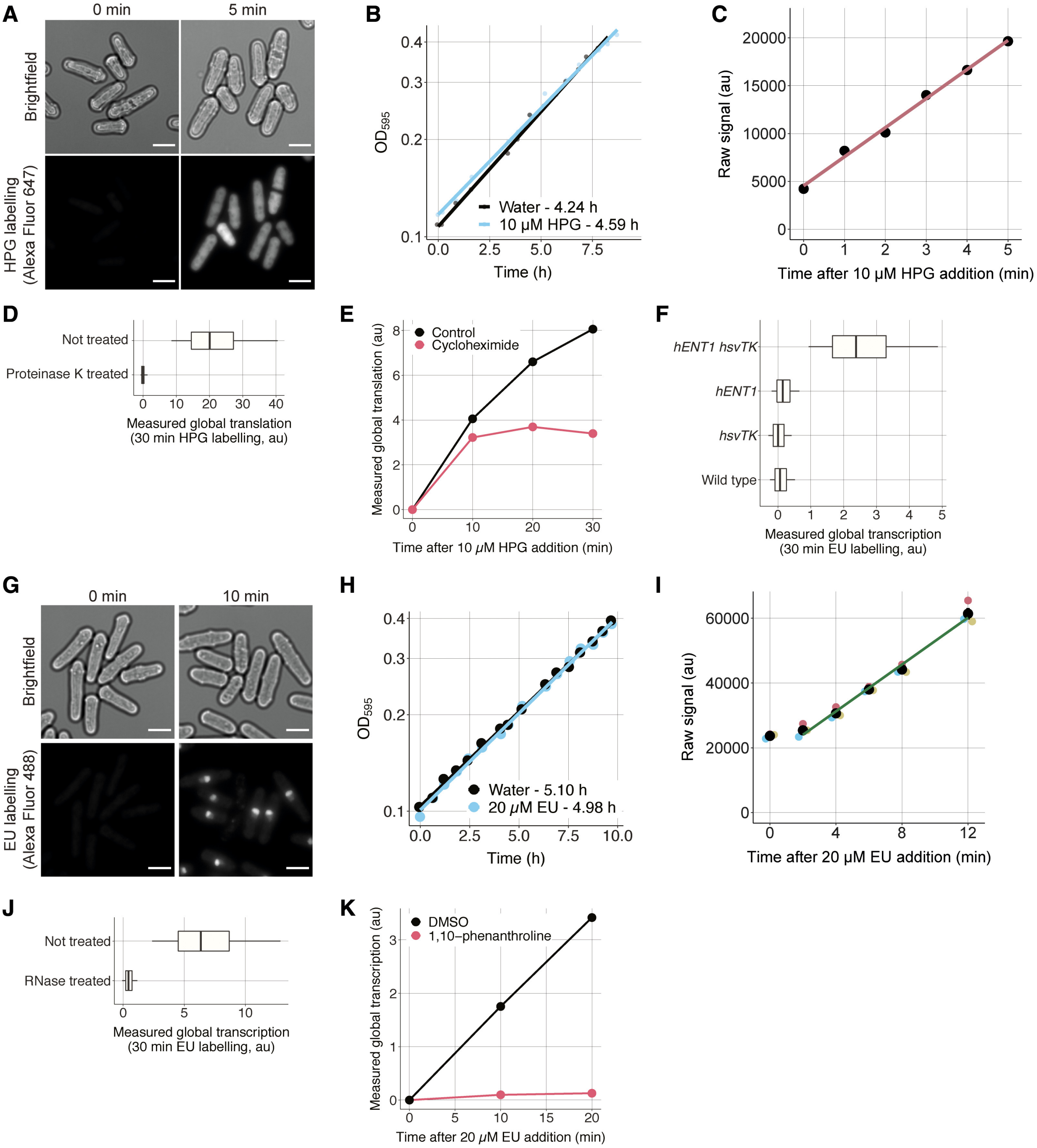
A
0 min
5 min
Brightfield
HPG labelling (Alexa Fluor 647)
B
OD595
Time (h)
Water - 4.24 h
10 μM HPG - 4.59 h
C
Raw signal (au)
Time after 10 μM HPG addition (min)
D
Not treated
Proteinase K treated
Measured global translation (30 min HPG labelling, au)
E
Control
Cycloheximide
Measured global translation (au)
Time after 10 μM HPG addition (min)
F
hENT1 hsvTK
hENT1
hsvTK
Wild type
Measured global transcription (30 min EU labelling, au)
G
0 min
10 min
Brightfield
EU labelling (Alexa Fluor 488)
H
OD595
Time (h)
Water - 5.10 h
20 μM EU - 4.98 h
I
Raw signal (au)
Time after 20 μM EU addition (min)
J
Not treated
RNase treated
Measured global transcription (30 min EU labelling, au)
K
DMSO
1,10–phenanthroline
Measured global transcription (au)
Time after 20 μM EU addition (min)

Figure EV1.

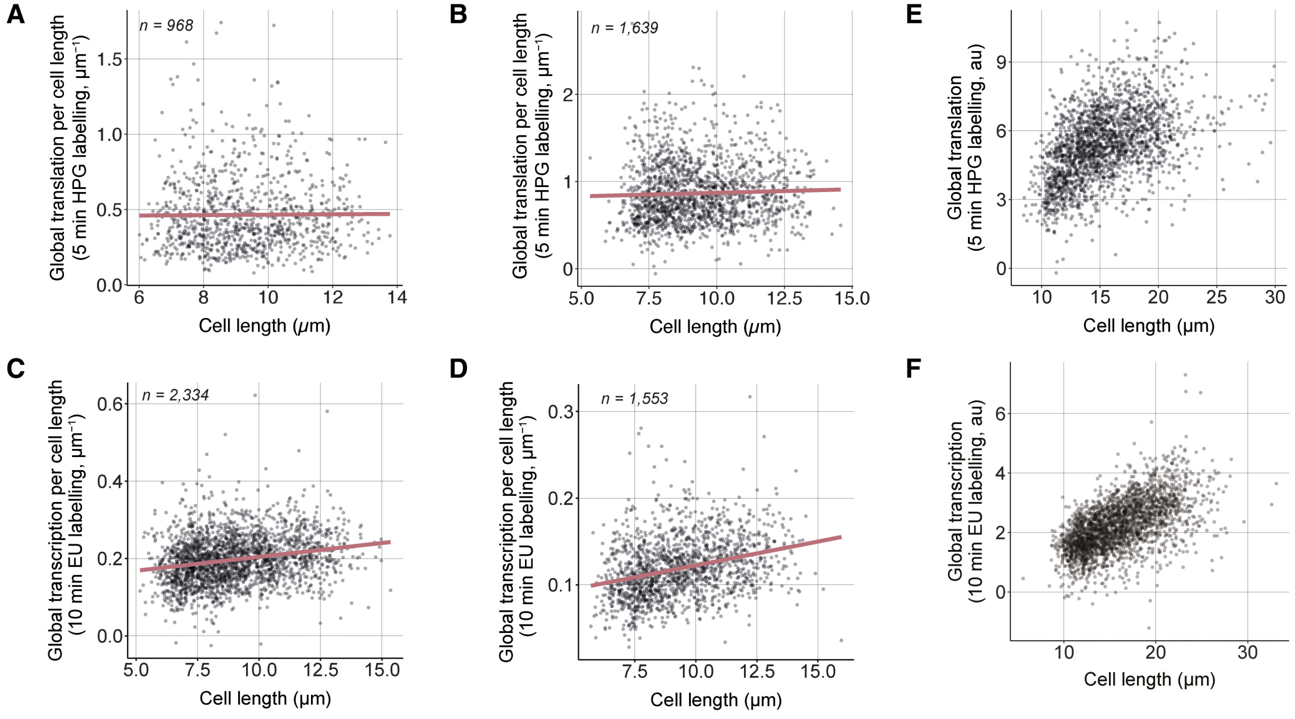

Figure EV2. Global transcription per size, but not global translation per size, somewhat increases with size.

A, B Experimental replicates of Fig 2C.

C, D Experimental replicates of Fig 2F.

E Global cellular translation of *cdc25-22* (PN143) single cells.

F Global cellular transcription of *hENT1 hsvTK cdc25-22* (PN5998) single cells.

 © 2023 The Francis Crick Institute

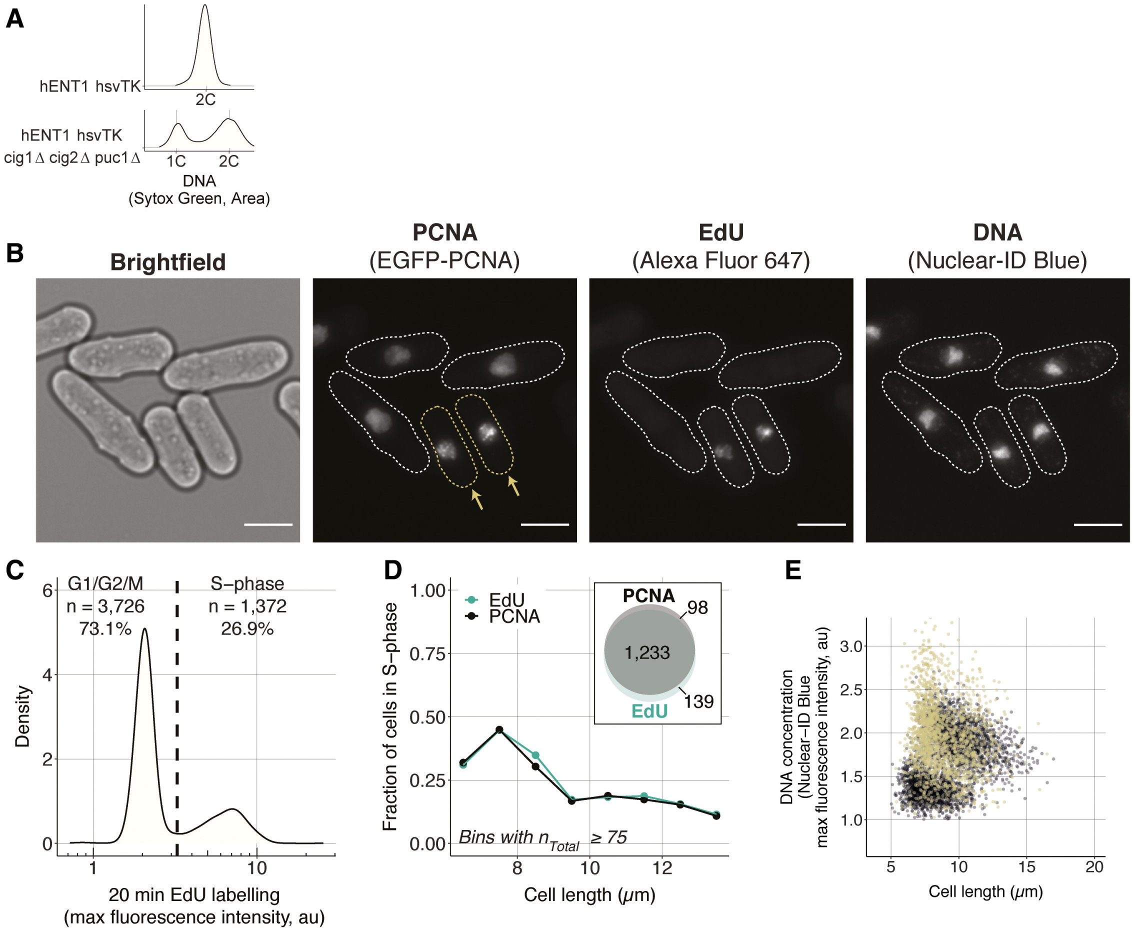

Figure EV3. The EGFP-PCNA marker allows for the accurate identification of cells in S-phase.

A Distribution of the amount of DNA in single cells in asynchronous populations, measured using the total fluorescence signal of Sytox Green by flow cytometry. For both populations, more than 200,000 cells were measured. Note that the 2C peak of the CCPΔ population (PN5999) is shifted to the right because the cells are longer and therefore have more mitochondrial DNA than the non-delete strain (PN10597).

B *hENT1 hsvTK EGFP-pcn1 CCPΔ* cells (PN6000) were pulsed with 200 μM EdU for 20 min and EdU incorporated in replicated DNA was fluorescently labelled using the same staining procedure used in the global transcription assay. Cells with visible foci in the PCNA channel are highlighted and marked with yellow arrows. The dotted white lines in the PCNA, EdU and DNA channels delimit the cell masks generated from the bright-field image. The scale bar represents 5 μm.

C Distribution of maximum fluorescence intensity of cells labelled with EdU. The dashed line represents the threshold (3.25 au) above which cells are considered in S-phase.

D The fraction of cells in S-phase per cell length is computed using the EdU signal shown in (C), or the presence of EGFP-PCNA foci determined by eye. The inset shows the overlap in cell numbers between the two methods of identifying S-phase cells.

E Same as Fig 3H with the S-phase population added in yellow.

© 2023 The Francis Crick Institute

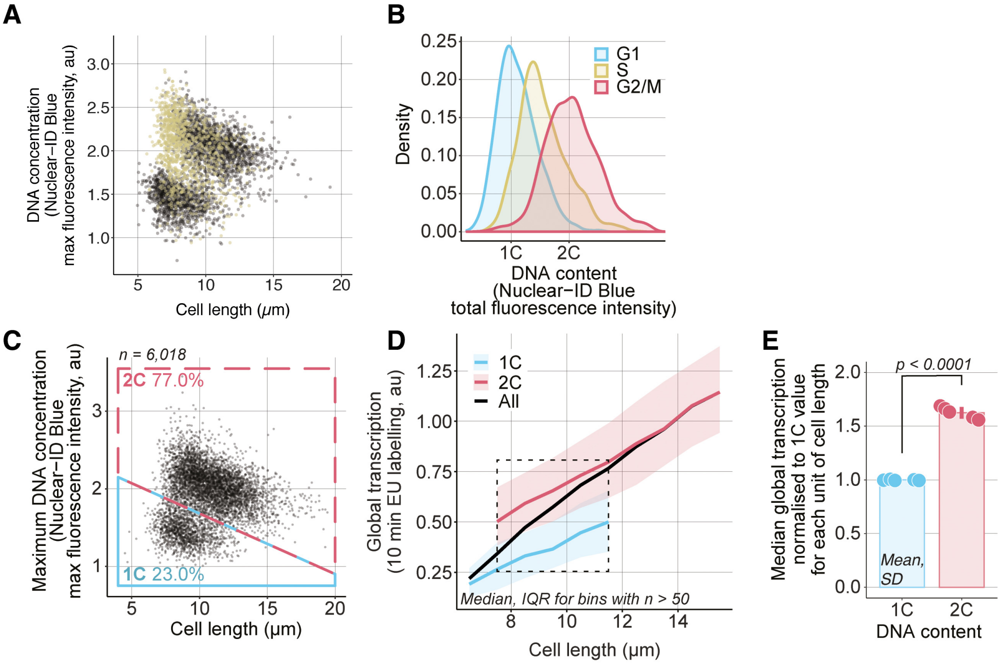

Figure EV4. A strain without the EGFP-PCNA marker also shows an increase in global transcription with cell length and from G1 to G2.

A Same as Fig 4B with the S-phase population added in yellow.

B Distribution of total DNA content of the cell populations categorised in Fig 4A and B as measured per total Nuclear-ID Blue fluorescence intensity per cell.

C *hENT1 hsvTK CCP*Δ (PN5999) cells were assayed for global transcription. The DNA concentration, measured as the maximum fluorescence intensity of the Nuclear-ID Blue stain in a cell, and cell length are used to categorise cells as having either 1C (blue box) or 2C DNA (red box). The percentage of cells in each box is shown. Black dots are single-cell measurements.

D Cells are grouped in bins of 1 μm. Medians (solid lines) and interquartile ranges (shaded areas) are shown for 1C (blue) or 2C DNA (red) populations. The dashed line box marks the length bins which have both a 1C and 2C median global transcription values.

E For each of the five length bins boxed in (B), both 1C and 2C medians are normalised to their respective global transcription 1C value. The normalised values are represented as dots, each dot corresponding to one of the five length bins, and the mean and SD of the normalised values are shown for each DNA content. The dots represent the median global transcription measurements per length bin boxed in (B). For each cell cycle stage, the normalised values (dots) are in the same order (left to right) as their corresponding length bins in (B). The *P*-value is calculated using Welch's unequal variances *t*-test.

© 2023 The Francis Crick Institute
