# Supplementary material for: The cell cycle and cell size influence the rates of global cellular translation and transcription in fission yeast
Source: EMBO J. 2023 Mar 23;42(9):e113333. doi: 10.15252/embj.2022113333 (PMC10152140; doi:10.15252/embj.2022113333)
Supplement: Supplementary file 2 — PDF+ [file EMBJ-42-e113333-s003.pdf]

Article

TRANSPARENT PROCESS OPEN ACCESS

THE EMBO JOURNAL

The cell cycle and cell size influence the rates of global cellular translation and transcription in fission yeast

Clovis Basier[1,*] & Paul Nurse[1,2]

Abstract

How the production of biomass is controlled as cells increase in size and proceed through the cell cycle events is important for understanding the regulation of global cellular growth. This has been studied for decades but has not yielded consistent results, probably due to perturbations induced by the synchronisation methods used in most previous studies. To avoid this problem, we have developed a system to analyse unperturbed exponentially growing populations of fission yeast cells. We generated thousands of fixed single-cell measurements of cell size, cell cycle stage and the levels of global cellular translation and transcription. We show that translation scales with size, and additionally, increases at late S-phase/early G2 and early in mitosis and decreases later in mitosis, suggesting that cell cycle controls are also operative over global cellular translation. Transcription increases with both size and the amount of DNA, suggesting that the level of transcription of a cell may be the result of a dynamic equilibrium between the number of RNA polymerases associating and disassociating from DNA.

Keywords cell cycle; cell size; scaling; transcription; translation
Subject Category Cell Cycle
DOI 10.15252/embj.2022113333 | Received 19 December 2022 | Revised 7 March 2023 | Accepted 8 March 2023 | Published online 23 March 2023
The EMBO Journal (2023) 42: e113333

Introduction

Proliferating steady-state eukaryotic cells undergo two fundamental processes: they increase in biomass and they undergo cycles of cell division. Biomass increase is a continuous process while the cell cycle consists of an orderly transition through a series of specific discrete events. How these continuous and punctuated processes impact on the accumulation of proteins and RNA, the major drivers of biomass increase, is important for understanding how overall cellular growth is regulated (Marguerat & Bähler, 2012). Proteins make up 35–60% and RNA 4–12% of the dry mass of cells (Feijó Delgado *et al*, 2013) and their production consumes more than half of the ATP of a cell (Buttgereit & Brandt, 1995). Previous studies of the patterns of protein and RNA synthesis through the cell cycle have led to conflicting results. In this paper, we have addressed this problem using unperturbed steady-state growing fission yeast cells.

Knowing the pattern of protein and RNA accumulation during the growth of cells through the cell cycle is an example of the general problem of scaling, a power-law relationship between two variables. The accurate scaling of protein and RNA synthesis with cell size maintains their concentration at a constant level, and there is evidence that loss of proper scaling of biosynthesis leads to cellular dysfunction and may be a causal driver for ageing and senescence (Neurohr *et al*, 2019; preprint: Cheng *et al*, 2021; preprint: Lanz *et al*, 2021; Lengefeld *et al*, 2021). Growth is continuous while cell cycle events are temporally discrete changes within cells. In particular, the amount of DNA, the template for RNA production, doubles once during S-phase early on in the cycle, and mitosis and cell division at the end of the cell cycle involve major cellular reconstruction. These can affect global cellular translation, the rate of synthesis of all proteins and global cellular transcription, the rate of synthesis of all RNA molecules.

Global cellular translation has been investigated in numerous cellular and experimental systems with varying outcomes. Studies using incorporation of exogenous amino acids to measure global translation in populations of synchronised yeasts have yielded conflicting results, either that global cellular translation undergoes significant changes during the cell cycle (Creanor *et al*, 1982) or remains constant (Elliott & Mclaughlin, 1978, 1979a,b; Stonyte *et al*, 2018). In mammalian cell cultures, studies monitoring global cellular translation through synchronised cell cycles were also contradictory, with some finding no changes (Coldwell *et al*, 2013) while others found increases and/or decreases of varying magnitudes during mitosis (Prescott & Bender, 1962; Konrad, 1963; Fan & Penman, 1970; Qin & Sarnow, 2004; Tanenbaum *et al*, 2015; Miettinen *et al*, 2019). Asynchronous cultures of yeasts and mammalian cells have not displayed major cell-cycle-related changes, suggesting that previous discrepancies may have been due to synchronisation methods (Stonyte *et al*, 2018). Previous studies of global cellular transcription through the cell cycle have relied on population

1 Cell Cycle Laboratory, The Francis Crick Institute, London, UK
2 Laboratory of Yeast Genetics and Cell Biology, Rockefeller University, New York, NY, USA
*Corresponding author. Tel: +44 02037961946; E-mail: clovis.basier@crick.ac.uk

© 2023 The Francis Crick Institute. Published under the terms of the CC BY 4.0 license

measurements of the incorporation into RNA of pulse-labelled nucleobases or nucleosides in synchronous cultures. In the two yeasts, *Schizosaccharomyces pombe* (Wain & Staatz, 1973; Fraser & Moreno, 1976; Fraser & Nurse, 1978, 1979; Elliott, 1983) and *Saccharomyces cerevisiae* (Fraser & Carter, 1976;Hynes & Phillips, 1976; Elliott & Mclaughlin, 1978, 1979a,b), these studies have also yielded variable results. Some studies found that RNA synthesis increased at a discrete stage of the cell cycle, either at DNA replication (Wain & Staatz, 1973; Fraser & Carter, 1976; Fraser & Moreno, 1976) or later (Fraser & Nurse, 1978, 1979; Elliott, 1983), while others found a constant increase throughout the cell cycle (Hynes & Phillips, 1976; Elliott & Mclaughlin, 1978, 1979a,b). Work in unperturbed mammalian cell lines suggests that global cellular transcription increases from G1 to G2 (Berry *et al*, 2022) and so these discrepancies may arise from the different protocols used to generate synchronous populations (Elliott, 1983). There may also be variations between organisms and cell types.

In this work, we characterise the scaling of global cellular translation and global cellular transcription in unperturbed cells during their growth and progression throughout their cell cycles. We have used the fission yeast, an organism that has been extensively used for cell cycle studies, taking single-cell approaches to investigate steady-state exponentially growing cells to avoid problems induced by cell-cycle synchronisation.

Results

Single-cell assays to measure global cellular translation and transcription in asynchronous steady-state exponentially growing cultures

To measure rates of global cellular translation and transcription through the cell cycle of fission yeast cells, we developed assays to quantify these rates while measuring cell size and identifying cell cycle stages in thousands of single cells in exponentially growing cultures. This is possible because fission yeast cells are rods that grow by tip elongation, so cell length is an indicator of cell cycle position (Mitchison, 1957).

To quantify global cellular translation, we incubated cells with a methionine analogue, L-homopropargylglycine (HPG), and measured its incorporation into proteins using Click Chemistry. Wild-type cells were incubated for 5 min with HPG and were stained with an Alexa Fluor azide (Figs 1A and B, and EV1A and B). The increase in HPG labelling had almost no lag and was essentially linear for a 5-min period after HPG addition (Fig 1C), indicating that a 5-min pulse could be used to estimate the rate of HPG incorporation. The pulse signal was five times the background signal (Fig EV1C). Digestion of protein molecules using proteinase K removed the fluorescent signal (Fig EV1D), and inhibiting translation using cycloheximide inhibited HPG incorporation (Fig EV1E). Thus, a 5-min HPG incubation and labelling can be used as a measure of global cellular translation. There is some HPG signal in the nucleus (Fig 1B), which is possibly the result of nuclear translation (David *et al*, 2012; Reid & Nicchitta, 2012; Stonyte *et al*, 2018) and/or rapid translocation into the nucleus of peptides synthesised in the cytoplasm.

To quantify global cellular transcription, we incubated cells with the uridine analogue, 5-ethynyluridine (EU), and measured its incorporation into all major RNA species (Jao & Salic, 2008) using Click Chemistry to fluorescently label EU molecules. EU was added to a culture of exponentially growing cells expressing the human equilibrative nucleoside transporter 1 (hENT1) and the herpes simplex virus thymidine kinase (hsvTK), necessary for the uptake and phosphorylation of EU (Fig EV1F; Sivakumar *et al*, 2004). After 10 min, cells were fixed, permeabilised and EU molecules were fluorescently labelled with an Alexa Fluor azide (Figs 1D and E and EV1G and H). The EU labelling signal was linear from 2 to 12 min (Fig 1F) indicating that a 10-min incubation could be used to estimate the rate of EU incorporation into RNA. However, in contrast to the translation assay, the transcription pulse signal was less strong and was only twice the background signal (Fig EV1I), which may result in a less accurate estimate of the rate of transcription. Linearity is also not much influenced by a longer 20-min incubation (Fig 1F), and was used in one experiment where incorporation was low (see later Fig 4). Digestion of RNA molecules using RNAse A removed the EU labelling signal (Fig EV1J), and inhibition of RNA polymerases using 1,10-phenanthronline inhibited EU incorporation (Fig EV1K). Thus, a 10/20-min EU incubation and labelling can be used as a measure of global cellular transcription.

To obtain single-cell measurements of cell size and global translation or transcription, we used bright-field and fluorescence microscopy in combination with automated segmentation tools (Berg *et al*, 2019). A fluorescent DNA dye (Nuclear-ID Blue) was used to identify and remove binucleated cells allowing analysis of a cell population in which all cells are composed of a single nucleus (Fig 1G).

Global cellular translation and transcription change with cell length

We used these two assays to investigate how global cellular translation and transcription are affected by cell size, and by progression through G2 within an asynchronous population (Fig 2A–F). We used cell length as a measurement of size, as cell length is correlated with cell volume because *S. pombe* cells are cylinders that grow by tip extension (Mitchison, 1957). Binucleated and septated cells were excluded from the analysis to eliminate the effects of mitosis and cell division, as well as S-phase which occurs during septation in wild-type cells (Moreno & Nurse, 1994). The median global translation increased smoothly with cell length (Fig 2A and B) and global translation per cell length was found to be constant with size (Figs 2C and EV2A and B). Thus, in the wild-type cells, global translation scales linearly with cell size as mononucleated cells proceed through the cell cycle.

Likewise, median global transcription increased smoothly with cell length in the *hENT1 hsvTK* population (Fig 2D and E), although global transcription per cell length increased somewhat as cell length increased during the cell cycle (Figs 2F and EV2C and D). Because the cells are growing in steady state, it would be expected that the rate per unit cell length at the end of the cell cycle would be the same as at the beginning of the cell cycle. We suggest that the increase we observed may be a consequence of the low signal-to-noise ratio of around 2:1 (Fig EV1I) leading to a technical defect in the background estimate. However, since global transcription increases smoothly with cell length (Fig 2E), we can conclude that the rise in transcription as cells grow does not exhibit any discontinuities.

© 2023 The Francis Crick Institute

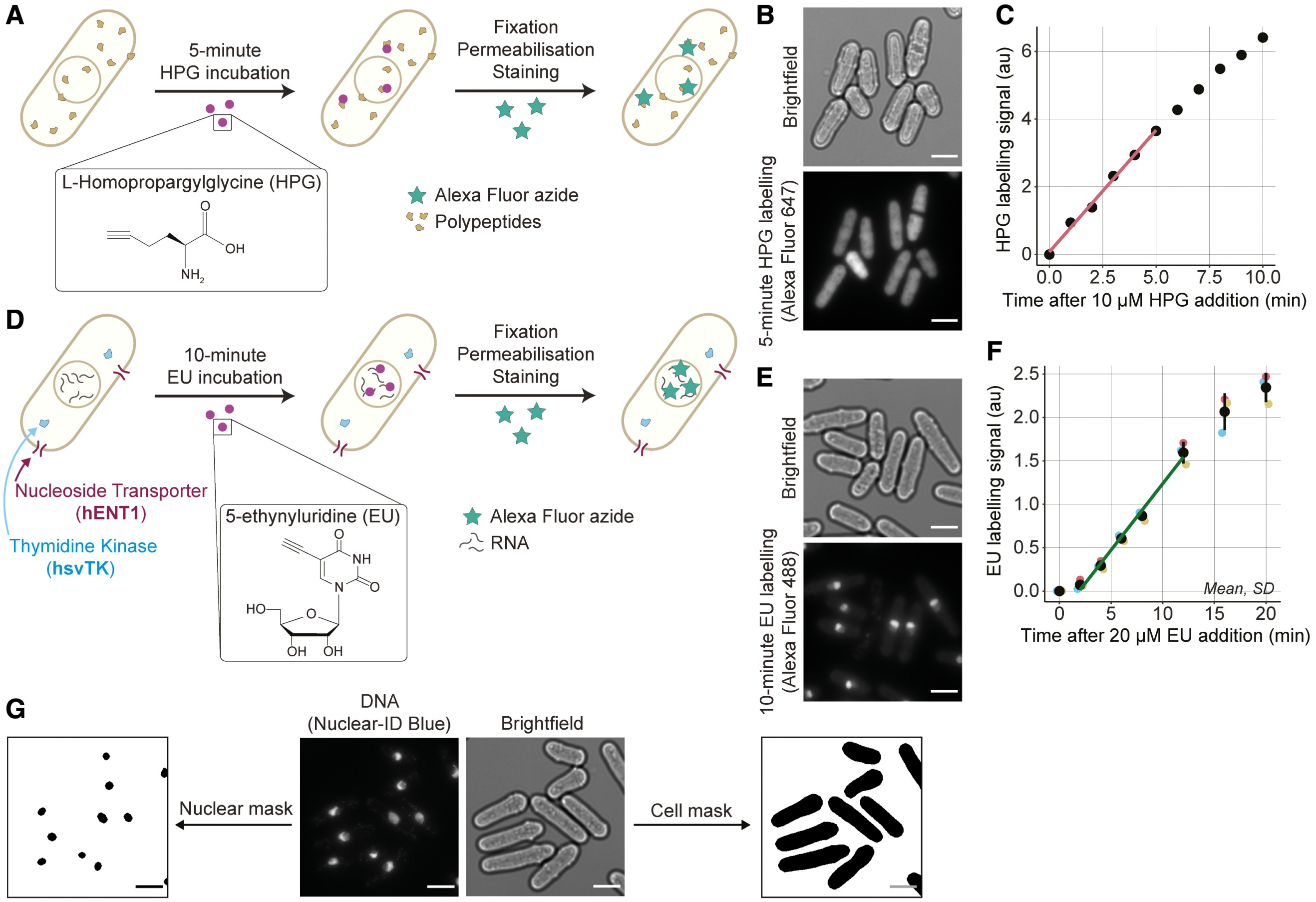

Figure 1. Single-cell assays to measure global cellular translation and transcription in steady-state growing asynchronous cultures.

A Overview of the global cellular translation assay. Wild-type cells are incubated with HPG for 5 min, then fixed, permeabilised and an Alexa Fluor azide fluorophore is covalently attached to HPG molecules using Click Chemistry.

B Example images of bright-field and fluorescently labelled HPG (Alexa Fluor 647) of wild-type cells (PN1) assayed for global cellular translation. Scale bars represent 5 μm.

C Change in HPG labelling signal with different durations of HPG incubation (in PN1), measured by flow cytometry. Population medians of at least 200,000 cells are shown. The red line is the ordinary least square (OLS) linear regression fitted on the medians between 0 and 5 min.

D Overview of the global cellular transcription assay. Cells expressing *hENT1* and *hsvTK* are incubated with EU for 10 min, then fixed, permeabilised and an Alexa Fluor azide fluorophore is covalently attached to EU molecules using Click Chemistry.

E Example images of bright-field and fluorescently labelled EU (Alexa Fluor 488) of *hENT1* and *hsvTK* cells (PN10567) assayed for global cellular transcription. Scale bars represent 5 μm.

F Change in EU labelling signal with different lengths of EU incubations, measured by flow cytometry (in PN10597). The mean and standard deviation (SD) of the population medians of at least 200,000 cells in experimental triplicates are shown in black. The dark green line is the OLS linear regression fitted on the mean data between 2 and 12 min.

G Example images of bright field (PN10597) used to generate cell masks, and DNA (Nuclear-ID Blue) used to generate nuclear masks. Scale bars represent 5 μm.

To investigate the effect of sizes beyond those of wild-type cells, we used the temperature-sensitive *cdc25-22* allele. When grown at a semi-permissive temperature, *cdc25-22* cells divide at longer lengths than wild-type cells while maintaining the same doubling time and not displaying any cell cycle defects (Fig 2G; Nurse *et al*, 1976; Zhurinsky *et al*, 2010). We found that global translation increased with cell length only in cells up to a length of 15 μm, a size approximately 10% more than the size at which wild-type cells divide. In cells longer than 15 μm, the rate of global translation reduced and then plateaued at lengths above about 19 μm (Figs 2H and I, and EV2E). In contrast, in an asynchronous population of *cdc25-22* *hENT1 hsvTK* cells grown at a semi-permissive temperature of 30°C, global transcription decreased only slightly with cell length up to lengths of 22 μm around 60% longer than dividing wild-type cells (Figs 2J and K, and EV2F). The decrease in transcription with cell length that we observe in the *cdc25-22 hENT1 hsvTK* (Fig 2K) cells but not in the *hENT1 hsvTK* cells (Fig 2F) might be due to the low signal-to-noise ratio. A decrease in transcription was reported in a population of enlarged fission yeast cells blocked in the cell cycle progression, but these cells were larger, being over twice the size of dividing wild-type cells (Zhurinsky *et al*, 2010). Therefore, the global transcription machinery is not saturated in cells up to 22 μm

© 2023 The Francis Crick Institute

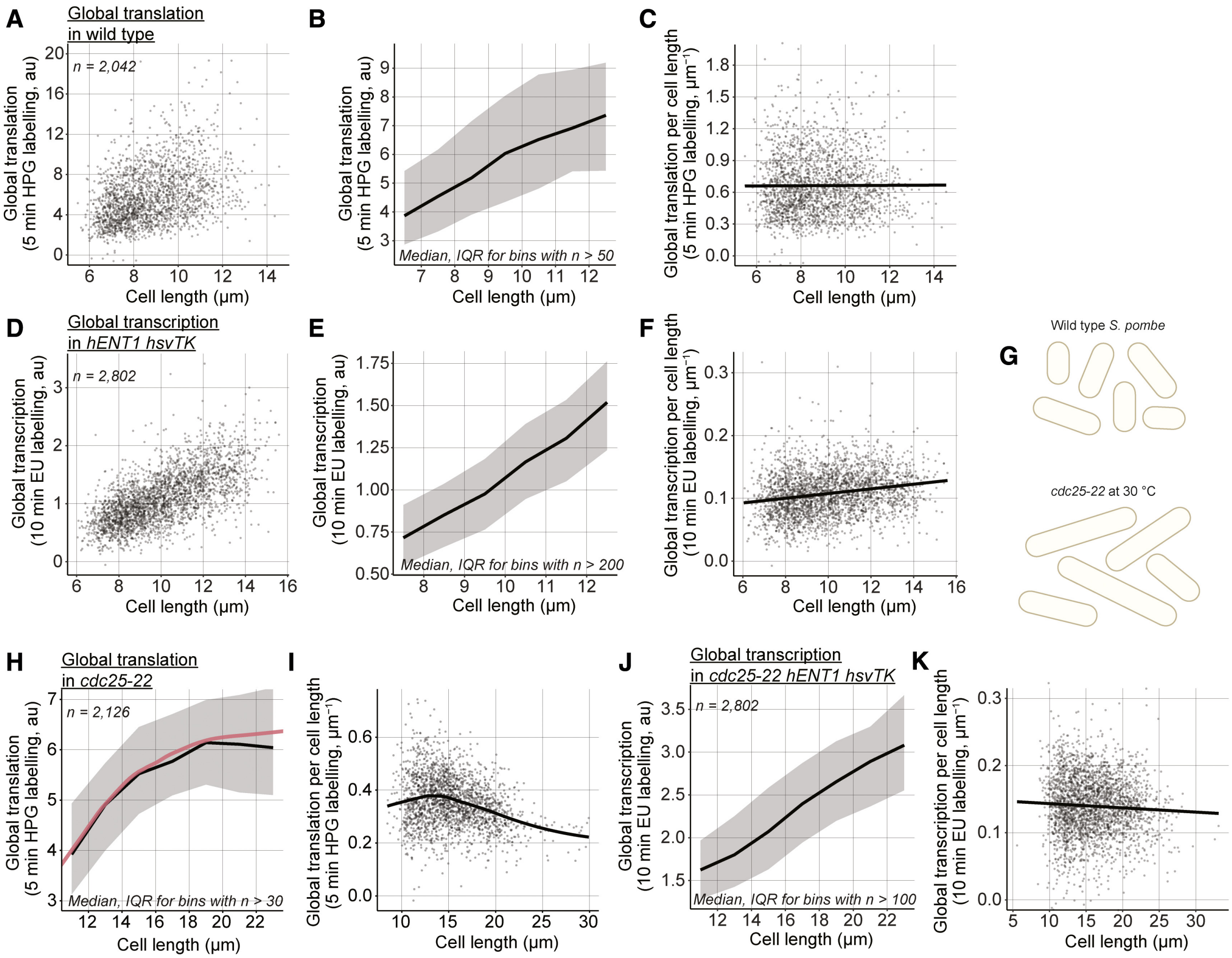

Figure 2. Global cellular translation and transcription with cell length in wild-type cells.

- A Global cellular translation of wild-type (PN1) single cells.
- B Medians of global translation (solid black line) and interquartile ranges (IQR, shaded area) of cells shown in (A) grouped in length bins of 1 µm. Bins containing more than 50 cells are shown.
- C Global cellular translation of cells shown in (A) divided by their cell length. The solid black line represents the OLS linear regression fitted on the data.
- D Global cellular transcription of single cells expressing *hENT1* and *hsvTK* (PN10597).
- E Medians of global transcription (solid black line) and IQR (shaded area) of cells shown in (D) grouped in length bins of 1 µm. Bins containing more than 200 cells are shown.
- F Global cellular transcription of cells shown in (D) divided by their cell length. The solid black line represents the OLS linear regression fitted on the data.
- G Schematic representation of cell length in asynchronous populations of the wild type and the *cdc25-22* mutant grown at the semi-permissive temperature (30°C).
- H Medians of global translation (solid black line) and IQR (shaded area) of *cdc25-22* (PN143) cells grown at 30°C and grouped in length bins of 2 µm. Bins containing more than 30 cells are shown. The solid red lines represent a locally estimated scatterplot smoothing (LOESS) function fitted on the single-cell data.
- I Global cellular translation of cells shown in (H) divided by their cell length. The solid black line represents the LOESS function fitted in (H).
- J Medians of global cellular transcription (solid black line) and IQR (shaded area) of *cdc25-22 hENT1 hsvTK* (PN5998) cells grown at 30°C and grouped in length bins of 2 µm. Bins containing more than 100 cells are shown.
- K Global cellular transcription of cells shown in (J) divided by their cell length. The solid black line represents the OLS linear regression fitted on the data.

while the rate of translation plateaus at 19 µm. We conclude that the plateau of global translation is unlikely to be due to transcription becoming limiting.

Global cellular translation from G1 to G2

We next sought to determine how global translation and transcription scale with the increase in the amount of DNA at S-phase and with the cellular changes happening as cells proceed through mitosis and cell division. Wild-type *S. pombe* cells spend the majority of their cell cycle in G2. The G1-phase is short so that DNA replication starts soon after completion of mitosis and is mostly completed by the time septated cells split to form two daughter cells (Moreno & Nurse, 1994). This is also the case for the *hENT1 hsvTK* strain (Fig EV3A). To assay protein and RNA synthesis in exponentially growing populations with cells of overlapping sizes in G1 and G2,

 © 2023 The Francis Crick Institute

and to assess cell cycle effects in cells of the same size, we used the *cig1Δ cig2Δ puc1Δ* (*CCPΔ*) strain. This mutant strain has a delayed and more variable onset of S-phase compared to wild-type cells (Martín-Castellanos *et al*, 2000; Figs 3A and EV3A). This means that the cell population has cells that are of the same size but which are located in the G1- or G2-phases of the cell cycle.

CCPΔ cells were assayed for global translation and their DNA was stained with Nuclear-ID Blue. The maximum DNA concentration was determined within each cell and was used to classify cells as having either 1C or 2C DNA content (Fig 3B). Cell lengths were also measured. In both the 1C and the 2C DNA content subpopulations, the median global translation per cell increased with cell size. The median global translation in cells of similar length increased by 35–40% in the G2 cells with a 2C DNA content compared with G1 cells with a 1C DNA content (Fig 3C and D).

To understand further the increase in global translation from G1 to G2, we identified the S-phase subpopulation using a strain-containing PCNA fused to an EGFP fluorescence marker (Meister *et al*, 2003). During S-phase, EGFP-PCNA forms foci on replicating DNA so that cells in S-phase can be identified using fluorescence microscopy (Meister *et al*, 2007; Fig 3E and F). The population of cells identified with EGFP-PCNA foci almost entirely overlapped with the population of cells replicating their DNA when assayed using 5-ethynyl-2′-deoxyuridine (EdU) (Fig EV3B–D), indicating that the presence of EGFP-PCNA foci reliably identifies S-phase cells. These *CCPΔ EGFP-pcn1* cells were assayed for global translation. We identified cells with EGFP-PCNA foci (Fig 3G) and classified the remaining cells as in G1 or G2/M based on their DNA concentration and cell length (Figs 3H and EV3E). The distributions of total fluorescence intensity per cell of Nuclear-ID Blue are similar to the distributions of DNA content in the three populations, indicating a reliable attribution of cell cycle stages (Fig 3I). Global translation was observed to increase with cell length in all subpopulations (Fig 3J). For a given cell length, global translation increased from the S to the G2/M subpopulation by about 30–35%, but by < 5% from the G1 to the S-phase subpopulation (Fig 3J and K). This indicates that on transition of cells from S to G2, there is about a one-third increase in the rate of translation.

Global cellular transcription from G1 to G2

To understand how changes related to the G1-, S- and G2-phases affect global transcription, we assayed *CCPΔ EGFP-pcn1 hENT1 hsvTK* cells for global transcription using a 20-min EU incubation to compensate for their lower signal production. We identified cells with EGFP-PCNA foci (Fig 4A) and classified the remaining cells as in G1 or G2/M based on their DNA concentration and cell length (Figs 4B and EV4A and B). Global transcription increased for a given cell length from the G1 to the G2/M subpopulation by around 30–35% and the S-phase subpopulation was found to have an intermediary global transcription value between the G1 and G2/M subpopulations with an increase of around 20–25% compared to the G1 subpopulation (Fig 4C and D). This indicates that global transcription increases through S-phase, increasing approximately with the amount of DNA. An increase in global transcription with cell length and from G1 to G2 was also observed in a strain without the EGFP-PCNA marker (Fig EV4C–E).

Thus, both global translation and global transcription increase from G1 to G2. Translation increases at the S/G2 transition or early in G2 and so is likely to be due to a subsequent cell cycle event dependent on S-phase, while transcription increases throughout S-phase as DNA content increases.

Global cellular translation and transcription at mitosis

Next, we determined the dynamics of global translation and transcription at mitosis. To identify mitotic cells, we used strains expressing synCut3-mCherry, a truncated version of the condensin subunit Cut3 fused to the mCherry fluorescent reporter (Patterson *et al*, 2021). The synCut3-mCherry fusion protein is localised in the cytoplasm through interphase and rapidly accumulates in the nucleus at mitotic onset before being exported back to the cytoplasm from anaphase A onwards (Patterson *et al*, 2021; Fig 5A and B). Thus, in an asynchronous population, the progression through mitosis of each cell can be assessed based on the localisation of the synCut3-mCherry fluorescence signal and the number of nuclei in the cell. Uninucleated and binucleated cells with low-nuclear synCut3-mCherry are in interphase, uninucleated cells with high levels of nuclear synCut3-mCherry are in mitosis between mitotic onset and anaphase A, and binucleated cells with high-nuclear synCut3 are post-anaphase A. We assayed global translation in a *synCut3-mCherry* population and classified uninucleated and binucleated cells as having high- or low-nuclear synCut3 using their mean and median synCut3-mCherry fluorescence intensity (Fig 5C). We observed changes associated with the progression of cells into and through mitosis. For a similar cell size, global translation increased around 10% early in mitosis and decreased by about 20% after anaphase A to below pre-mitotic levels (Fig 5D and E). Thus, global translation increases and then decreases as cells proceed through mitosis. In contrast, when we assayed global transcription in the *synCut3-mCherry hENT1 hsvTK* strain and categorised cells as uninucleated or binucleated and having either high- or low-nuclear synCut3-mCherry (Fig 5F), we found no change in global transcription for a given cell length in the different mitotic subpopulations (Fig 5G). This suggests that global transcription is not affected by the cellular changes happening in mitosis.

Discussion

The rate of global transcription and to a lesser extent of translation have been investigated during the cell cycle of various eukaryotes (Wain & Staatz, 1973; Fraser & Carter, 1976; Fraser & Moreno, 1976; Hynes & Phillips, 1976; Elliott & Mclaughlin, 1978, 1979a,b; Fraser & Nurse, 1978, 1979; Elliott, 1983; Schmidt & Schibler, 1995; Zhurinsky *et al*, 2010), but the outcomes of these experimental investigations have been inconsistent with one another. This is probably due to effects of the different methods of synchronisation, perturbations due to a lack of steady-state growth and possible variations between organisms and cell types. In this work, we use a single-cell approach to generate thousands of measurements of cell size, cell cycle stage and global cellular translation and transcription, primarily investigating unperturbed, steady-state, exponentially growing fission yeast cells.

© 2023 The Francis Crick Institute

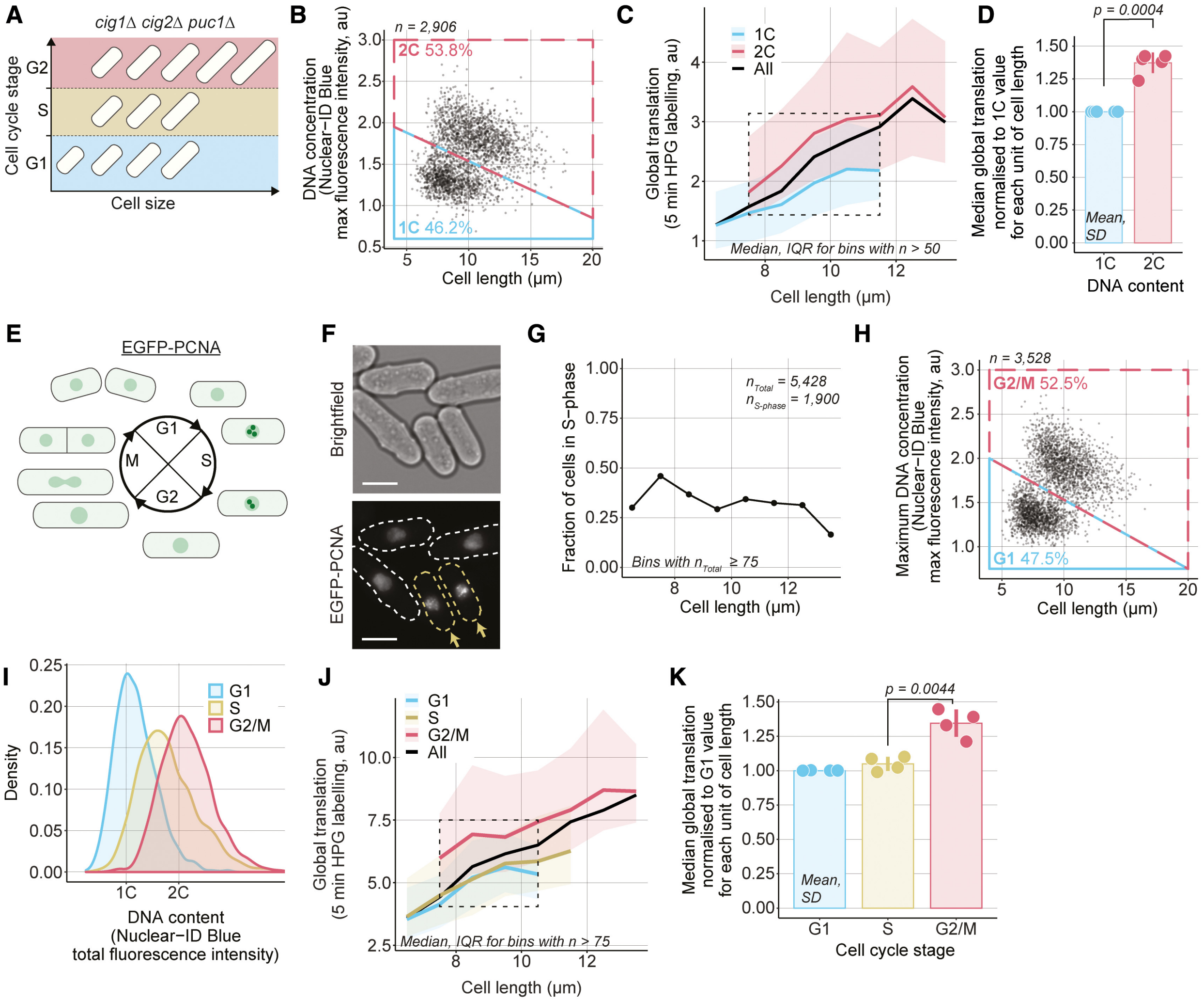

Figure 3. Global cellular translation from G1 to G2 in *CCP*Δ cells.

A Schematic representation of the G1, S and G2 subpopulation with overlapping cell sizes in the *CCP*Δ strain.

B *CCP*Δ (PN5792) cells were assayed for global cellular translation. The maximum DNA concentration, measured as the maximum fluorescence intensity of the Nuclear-ID Blue stain in a cell, and cell length are used to categorise cells as having either 1C (blue box) or 2C DNA (red box). The percentage of cells in each category is shown.

C Cells shown in (B) are grouped in length bins of 1 μm. Medians of global cellular translation (solid lines) and IQR (shaded areas) are shown for 1C (blue) and 2C DNA (red) subpopulations. The dashed line box marks the length bins which have both a 1C and a 2C median global cellular translation values. Bins containing more than 50 cells are shown.

D For each of the five length bins boxed in (C), both 1C and 2C medians are normalised to their respective 1C global cellular translation values. The normalised values are represented as dots, each dot corresponding to one of the five length bins, the mean and SD of the normalised values are shown for each DNA content. For each DNA content, the normalised values (dots) are in the same order (left to right) as their corresponding length bins in (C). The *P*-value is calculated using Welch's unequal variances *t*-test.

E Schematic of EGFP-PCNA localisation through the cell cycle.

F Example images of bright field and EGFP-PCNA fluorescence in *CCP*Δ *EGFP-pcn1* cells (PN6001). The dashed lines in the EGFP-PCNA channel delimit the cell masks generated from the bright-field image. Cells with visible foci in the EGFP-PCNA channel are highlighted in yellow and marked with arrows. Scale bars represent 5 μm.

G *CCP*Δ *EGFP-pcn1* cells (PN6001) were assayed for global cellular translation using a 5-min HPG incubation. Cells with EGFP-PCNA foci were identified by eye and binned in 1 μm intervals to compute the fraction of cells in S-phase per cell length.

H The maximum local DNA concentration (the maximum fluorescence intensity of the Nuclear-ID Blue DNA stain within a cell) and cell length were used to categorise cells not identified as in S-phase in (G) has either in G1 (blue box) or G2/M (red box). The percentage of cells in each category is shown.

I Distribution of total DNA content of the cell populations categorised in (G) and (H) as measured per total Nuclear-ID Blue fluorescence intensity per cell.

J Cells shown in (G) and (H) are grouped in length bins of 1 μm. Medians of global cellular translation (solid lines) and IQR (shaded areas) are shown for G1 (blue), S (yellow) and G2/M (red) subpopulations. The dashed line box marks the length bins which have G1, S and G2/M median global cellular translation values. Bins containing more than 75 cells are shown.

K For each of the four length bins boxed in (J), G1, S and G2/M medians are normalised to their respective G1 global cellular translation value. The normalised values are represented as dots, each dot corresponding to one of the four length bins, and the mean and SD of the normalised values are shown for each cell cycle stage. For each cell cycle stage, the normalised values (dots) are in the same order (left to right) as their corresponding length bins in (J). The *P*-value is calculated using Welch's unequal variances *t*-test.

© 2023 The Francis Crick Institute

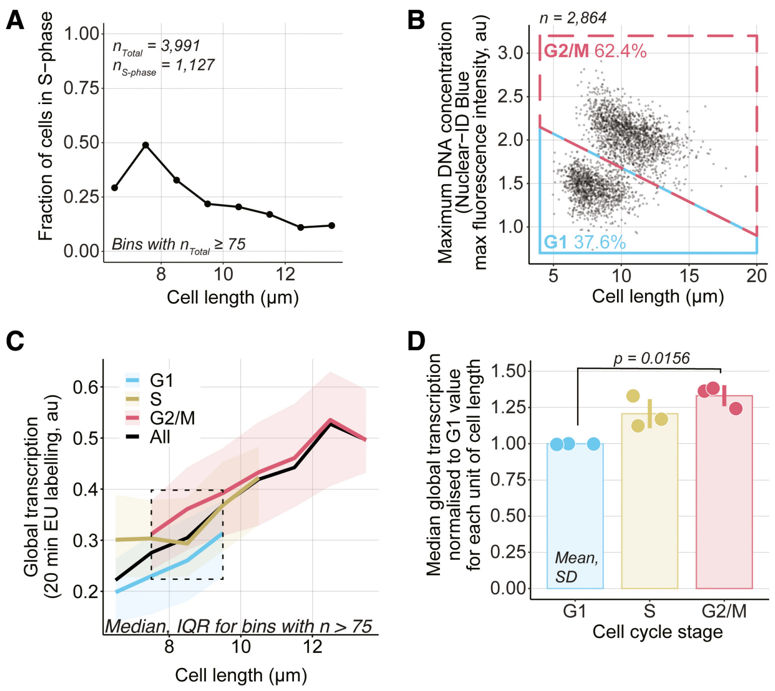

Figure 4. Global cellular transcription from G1 to G2.

A *hENT1 hsυTK CCPΔ EGFP-pcn1* cells (PN6000) were assayed for global transcription using a 20-min (almost linear, 15% deviation from the OLS linear regression shown in Fig 1F) EU incubation. Cells with EGFP-PCNA foci were identified by eye and binned on 1 μm intervals to compute the fraction of cells in S-phase per cell length.

B The maximum DNA concentration and cell length were used to categorise cells not identified as in S-phase in (A) as either in G1 (blue box) or G2/M (red box). The percentage of cells in each category is shown.

C Cells shown in (A) and (B) are grouped in length bins of 1 μm. Medians of global cellular transcription (solid lines) and IQR (shaded areas) are shown for G1 (blue), S (yellow) and G2/M (red) subpopulations. The dashed line box marks the length bins which have G1, S and G2/M median global transcription values. Bins containing more than 75 cells are shown.

D For each of the three length bins boxed in (C), G1, S and G2/M medians are normalised to their respective G1 global transcription value. The normalised values are represented as dots, each dot corresponding to one of the three length bins, and the mean and SD of the normalised values are shown for each cell cycle stage. For each cell cycle stage, the normalised values (dots) are in the same order (left to right) as their corresponding length bins in (C). The *P*-value is calculated using Welch's unequal variances *t*-test.

The rate of global cellular translation increases linearly with cell size in wild-type cells but plateaus at larger sizes. It is unclear what factor(s) may become limiting for global cellular translation in these larger cells. However, since global cellular transcription increases with cell size but does not plateau within the range of sizes assayed, the plateau in global cellular translation is unlikely to be due to RNA becoming limiting. This is consistent with previous work suggesting that growth is mainly driven by the number of active ribosomes in cells (Scott *et al*, 2010; Metzl-Raz *et al*, 2017) and that cells enlarged beyond wild-type sizes using cell cycle arrests experience cytoplasmic dilution of their proteins (Neurohr *et al*, 2019). The increase in global cellular translation at the S/G2 transition and the beginning of mitosis and the decrease later in mitosis suggest that there is regulation of global cellular translation as cells proceed through the events of the cell cycle. This is consistent with work in synchronised mammalian cells showing an increase in translation early in mitosis followed by a decrease later (Miettinen *et al*, 2019), and with work in asynchronous cultures of fission yeast grown in minimal medium containing isoleucine as a nitrogen source which showed no increase from G1 to S and an increase from S to G2 (Stonyte *et al*, 2018). In addition, our assay allows the identification of different stages of mitosis which was not previously possible in Stonyte *et al*, and shows that global translation follows the same increase and decrease patterns observed in mammalian cells (Miettinen *et al*, 2019). Interestingly, proteins involved in translation initiation have been identified as substrates of the fission yeast cyclin-dependant kinase (CDK1) Cdc2 (Swaffer *et al*, 2016) and CDK1 has been shown to phosphorylate the eukaryotic initiation factor 4E-binding protein (4E-BP1) in mammalian cell cultures (Shuda *et al*, 2015).

The rate of global cellular transcription increases with cell size in both wild-type cells, and in *cdc25-22* mutant cells which are up to 60% larger, and the rate of transcription is increased in cells undergoing S-phase by 20% compared to G1 cells and is 35% higher in G2 cells which have completed S-phase compared to G1 cells, indicating that DNA content is limiting the global rate of transcription. Previous work has suggested that transcription by one of the RNA polymerases Pol II increases with cell size (Padovan-Merhar *et al*, 2015; Sun *et al*, 2020; preprint: Swaffer *et al*, 2021). This, in addition to the fact that global cellular transcription does not plateau in *cdc25-22* cells which are up to 60% larger than wild-type cells, suggests that Pol II and other RNA polymerases are not saturated in these enlarged cells. Therefore, the increase in global cellular

© 2023 The Francis Crick Institute

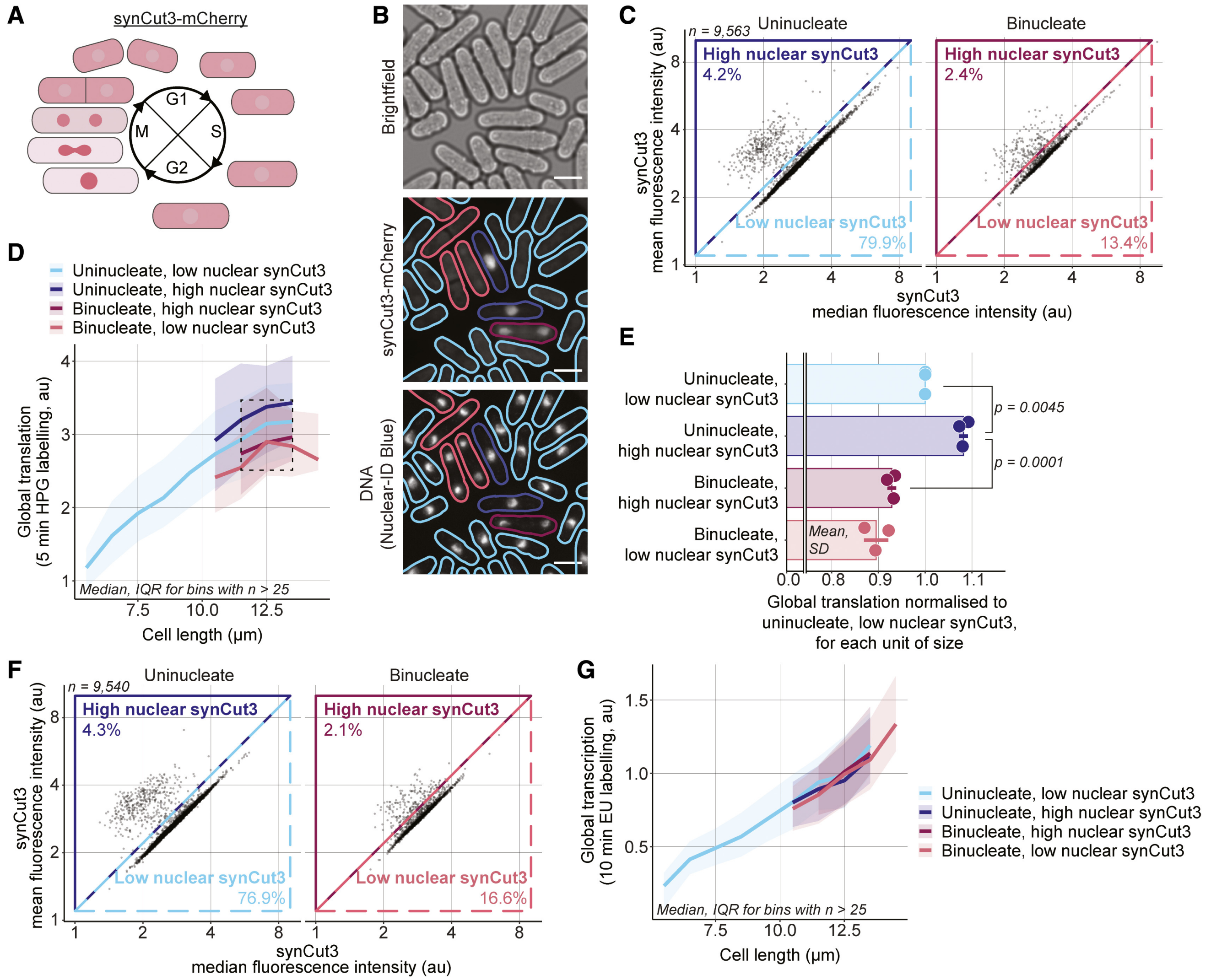

Figure 5. Global cellular translation and transcription at mitosis.

A Schematic of synCut3-mCherry localisation through the cell cycle.

B Example images of bright field, synCut3-mCherry, DNA (Nuclear-ID Blue) fluorescence in *synCut3-mCherry* cells (PN6004). The solid lines in the synCut3-mCherry and DNA channels delimit the cell masks generated from the bright-field image and are coloured according to the classification used in (D). The outlines of uninucleates are blue, binucleates are red, cells with high-nuclear synCut3-mCherry have dark outlines and cells with low-nuclear synCut3-mCherry have light outlines. Scale bars represent 5 μm.

C A population of *synCut3-mCherry* cells (PN6004) was assayed for global cellular translation. The mean and median whole-cell fluorescence synCut3-mCherry intensities of uninucleates and binucleates were used to categorise cells as having low-/high-nuclear synCut3-mCherry. The lines represent the delimitation of the different categories and the percentage of cells from the total population in each category is shown.

D Cells shown in (C) are grouped in length bins of 1 μm. Medians of global cellular translation (solid lines) and IQR (shaded areas) are shown for uninucleates (blue) and binucleates (red), and with low (light) and high (dark) nuclear synCut3-mCherry signal subpopulations. The dashed line box marks the length bins which have median global cellular translation values for all four subpopulations. Bins containing more than 25 cells are shown.

E For each of the three length bins boxed in (D), the medians of each subpopulation are normalised to their respective "uninucleate, low nuclear synCut3-mCherry" global cellular translation value. The normalised values are represented as dots, each dot corresponding to one of the three length bins, and the mean and SD of the normalised values are shown for each cell cycle stage. For each mitotic stage, the normalised values (dots) are in the same order (left to right) as their corresponding length bins in (D). The *P*-value is calculated using Welch's unequal variances *t*-test. For visual clarity, synCut3-mCherry is shortened to synCut3 in fig.

F Same as (C) for *synCut3-mCherry hENT1 hsvTK* cells (PN6005) assayed for global transcription.

G Cells shown in (F) are grouped in length bins of 1 μm. Medians of global cellular transcription (solid lines) and IQR (shaded areas) are shown for uninucleates (blue) and binucleates (red), and with low- (light) and high (dark)-nuclear synCut3-mCherry signal subpopulations. Bins containing more than 25 cells are shown.

transcription we observe from G1, through S-phase to G2, is unlikely to be the result of an increase in the amount of saturated DNA, but rather the result of an increase in the amount of unsaturated DNA leading to an increase in the probability of association of RNA polymerases with DNA. This is consistent with the dynamic equilibrium model for Pol II proposed for budding yeast

© 2023 The Francis Crick Institute

(preprint: Swaffer *et al*, 2021). This model assumes that the increase in the occupancy of RNA polymerases is due to a dynamic equilibrium between free polymerases associating with the DNA and detaching from the DNA. It is also possible that the increase happens at a certain stage of S-phase independent of the amount of DNA since we do not know the extent of S-phase of each cell. We did not observe a reduction in global cellular transcription through mitosis, unlike previous work in mammalian cells (Wansink *et al*, 1993; Gébrane-Younès *et al*, 1997) and budding yeast (Clemente-Blanco *et al*, 2009). It is possible that undertaking mitosis without breaking down the nuclear envelope (Zheng *et al*, 2007) prevents the reduction in transcription observed in mammalian cells undertaking open mitosis. Another explanation might be that the larger genome of mammalian cells undergoes greater condensation for longer than is the case for fission yeast. Budding yeast also has a closed mitosis and a small genome, but the work carried out in this organism was done using synchronised populations of cells so the difference observed might be the outcome of a perturbation as a consequence of synchronisation.

The cell cycle stage of a fission yeast cell and the activity of its CDK molecules are intrinsically linked since CDK activity defines the cell cycle stage of a cell. CDK activity increases through the cell cycle and is responsible for cells progressing through G1, S, G2 and mitosis (Coudreuse & Nurse, 2010; Swaffer *et al*, 2016) so that an unperturbed asynchronous population of cells in G1 is achieved by a low CDK activity. Thus, our results reflect changes happening through the cell cycle as the CDK regulation network undergoes modifications, and in steady-state cells, the cell cycle cannot be uncoupled from CDK activity. It is possible, however, that in the absence of the cyclins Cig1, Cig2 and Puc1, cell cycle transitions, particularly the G1/S transition, could have some differences compared with the wild type.

We propose that for the fission yeast, both translation and transcription steadily increase with cell size, but that the rate of translation becomes rapidly restricted when cells become larger than wild-type dividing cells. This suggests that a component or components required for translation become limiting. It is unlikely that synthesis of RNA is the limiting factor since transcription still increases with size in cells larger than the wild type while translation does not. This may be related to the large resource and energy requirements of protein synthesis, resulting in there being only limited capacity for continued increase in the rate of translation. In cells dividing at wild-type cell lengths, translation is regulated at different stages of the cell cycle; positively at the S/G2 transition and early in mitosis, and negatively later in mitosis. We hypothesise that changes in CDK activity through the cell cycle could influence the fraction of active ribosomes and be responsible for the cell-cycle-related changes in translation. Although the rate of transcription does not appear to be limiting in cells of this size, it is limited by DNA content. We suggest that global transcription is regulated by RNA polymerases which operate in dynamic equilibrium with DNA (preprint: Swaffer *et al*, 2021), and that global cellular translation is positively regulated in G2 possibly to coordinate with the increase in global cellular transcription that occurs during DNA replication. Global transcription and translation increase with cell size possibly exponentially, but the changes in global translation during transitions through cell cycle stages suggest that the rate of growth is modulated by cell cycle progression, increasing between S and G2 and early in mitosis and slowing down later in mitosis.

Previous studies of global cellular translation and transcription during the cell cycle have given conflicting results. Our single-cell approach gives us confidence that we have accurately described the changes of both translation and transcription with increasing cell size and progression through S-phase and mitosis/cell division in fission yeast cells. Knowledge of these changes is important for thinking about cellular control of macromolecular synthesis, and cell growth importance for the overall increase in cellular biomass. The approach we have used is employable with other eukaryotes to determine if there are conserved principles operating on these global cellular controls.

Materials and Methods

Reagents and Tools table

Reagent of resource	Source	Identifier
**Chemicals**		
EMM	MP Biomedicals	Cat# 114110012-CF
5-Ethynyluridine	Thermo Fisher	Cat# E10345
L-Homopropargylglycine	Cambridge Bioscience	Cat# 11785-50mg-CAY
16% Formaldehyde (w/v)	Thermo Fisher	Cat#11586711
NUCLEAR-ID Blue DNA stain	Enzo Life Sciences	Cat#ENZ-CHM103-0200
BSA	Sigma-Aldrich	Cat#A7906-100G
Triton X-100	Sigma-Aldrich	Cat#T9284-100ML
PBS	Gibco	Cat#11594516
Sodium citrate	Fisher Bioreagents	Cat#BP327-500
**Critical commercial assays**		
Click-iT Plus Alexa Fluor 488 Picolyl Azide Toolkit	Thermo Fisher	Cat# C10641
Click-iT Plus Alexa Fluor 647 Picolyl Azide Toolkit	Thermo Fisher	Cat# C10643

© 2023 The Francis Crick Institute

Reagents and Tools table (continued)

Reagent of resource	Source	Identifier
**Experimental models: organisms/strains**		
S. pombe: h- 972wt	Lab collection	PN1
S. pombe: h- cdc25-22	Lab collection	PN143
S. pombe: h- leu1-32::pFS181[adh1-hENT1 leu1+] pJL218[adh1-hsvTK his7+]	Lab collection	PN10597
S. pombe: h- cig1Δ::ura4+ cig2Δ::ura4+ puc1Δ::ura4+ura4-D18	Lab collection	PN5792
S. pombe: h- cdc25-22 leu1-32::pFS181[adh1-hENT1 leu1+] pJL218[adh1-hsvTK his7+]	This paper	PN5998 (CB41)
S. pombe: h- cig1Δ::ura4+ cig2Δ::ura4+ puc1Δ::ura4+ leu1-32::pFS181[adh1-hENT1 leu1+] pJL218[adh1-hsvTK his7+]	This paper	PN5999 (CB49)
S. pombe: h- ura4::pSMUG[EGFP-pcn1] cig1Δ::ura4+ cig2Δ:: ura4+ puc1Δ::ura4+ leu1-32::pFS181[adh1-hENT1 leu1] pJL218[adh1-hsvTK his7+]	This paper	PN6000 (CB71)
S. pombe: ura4::pSMUG[EGFP-pcn1] cig1Δ::ura4+ cig2Δ::ura4+ puc1Δ::ura4+	This paper	PN6001 (CB80)
S. pombe: h+ leu1-32::pFS181[adh1-hENT1 leu1+]	This paper	PN6002 (CB94)
S. pombe: h- pJL218[adh1-hsvTK his7+]	This paper	PN6003 (CB96)
S. pombe: h+ leu1-32::pNK05 [eno276P-synCut3-mCherry-adh1T leu1+]	This paper	PN6004 (CB117)
S. pombe: leu1-32::pFS181[adh1-hENT1 leu1+] pJL218[adh1-hsvTK his7+] [III,114483]::pNK05[eno276P-synCut3-mCherry-adh1T hphMX6]	This paper	PN6005 (CB135)
**Recombinant DNA**		
Plasmid: eno276P-synCut3-mCherry-adh1T hphMX6	Nitin Kapadia	pNK05
**Oligonucleotides**		
Tctgatttaaggatacgtagaactgcggtgag ttttccttgtgatctattatattacaatacacgggt tgtataagtagcCTCTTGCCCCTTCTAAGCTC	This work	oCB132
Ctcgttcctcagttcagttatgagctatattagtg ataggtaacattataacccagttaatacaatac ctatactcagttTATAGCGACCAGCATTCACA	This work	oCB133
**Software and algorithms**		
FACSDiva v8.0.1	BD	https://www.bdbiosciences.com/en-eu/products/software/instrument-software/bd-facsdiva-software#Overview
FIJI (ImageJ) v2.1.0/1.53c	NIH	https://fiji.sc/
Ilastik v1.3.0-OSX	Berg *et al* (2019)	https://www.ilastik.org/
R v4.1.0	R Core Team	https://www.r-project.org/
RStudio v1.4.1106	Team RStudio	https://www.rstudio.com/
Micro-Manager v2.0	Laboratory for Optical and Computational Instrumentation at the University of Wisconsin, Madison	https://micro-manager.org/

Methods and Protocols

Strain construction

All strains were constructed using random spore analysis after a genetic cross except for CB135 which was obtained by lithium acetate transformation of PN10597 with the [eno276P-synCut3-mCherry-adh1T hphMX6] construct from pNK05 amplified using the primers oCB132 and oCB133. All genotypes were confirmed phenotypically when possible (for temperature-sensitive alleles and fluorescent markers) or by PCR for gene deletions.

Cell cultures

Stationary cultures frozen and stored at −80°C in 50% (v/v) YFM are patched on YE agar plates and incubated overnight (O/N) at 32°C (or 25°C if temperature sensitive). The patch is then streaked on a fresh EMM agar plate and cells are grown at 25°C until visible

 © 2023 The Francis Crick Institute

single colonies form (typically around 4 days); a single colony is then patched on a fresh EMM agar plate and grown O/N at 25°C. A 5 ml EMM liquid culture is inoculated from a patch and grown in a stationary incubator O/N. The culture is then diluted in the morning in EMM to OD_{595} = 0.05 (calculated using an Amersham Ultraspec 2100 pro) in a flask and incubated for the day at 25°C in a shaking incubator. The culture is then diluted in EMM to OD595 = 0.025 and grown O/N in a flask at 25°C in a shaking incubator. In the morning, cells are diluted and used for the experiment.

Global cellular transcription assay

An exponentially growing *S. pombe* culture of *hENT1 hsvTK* cells in EMM at 25°C is diluted to 20 ml at OD595 = 0.3 (calculated using an Amersham Ultraspec 2100 pro) in a 50 ml flask, and placed in a shaking water bath for 1 h. Next, 4 μl of EU is added to the culture from a 100 mM stock solution in Milli-Q water to a final concentration of 20 μM. Immediately after the addition of EU, a 3.84 ml sample of the culture is taken and fixed with 1.16 ml of a stock solution of 16% (w/v) formaldehyde (methanol-free) in a 15 ml centrifuge tube, to a final concentration of 3.7%, and vortexed for 5 s before being incubated at room temperature (19–23°C) on a rocker, in the dark, for 40 min. This first sample will be used to compute the background signal. After 10 min, a second sample is taken from the culture and processed the same way, apart from being incubated for only 30 min. Fixed cells are then spun at 2,000 rcf for 5 min, and the supernatant is discarded. Cells are resuspended in 3 ml of PBS + 1% (w/v) BSA, vortexed for 5 s, spun at 2,000 rcf for 5 min and the supernatant is discarded. Cells are resuspended in 6 ml of PBS + 1% (w/v) BSA + 1% (v/v) Triton X-100, vortexed for 5 s and incubated at room temperature on a rocker for 30 min, in the dark. Cells are spun at 2,000 rcf for 5 min, the supernatant is discarded and cells are resuspended in 6 ml of PBS + 1% (w/v) BSA, vortexed for 5 s and incubated at room temperature on a rocker for 60 min, in the dark. Cells are spun at 2,000 rcf for 5 min, the supernatant is discarded, cells are resuspended in 500 of 1× Click-iT reaction buffer (Thermo Click-iT Plus picolyl azide kit) and transferred to a 1.5 ml centrifuge tube. Cells are spun at 2,000 rcf for 5 min, the supernatant is discarded and resuspended in 500 μl of the following reaction mix from the Thermo Click-iT Plus picolyl azide kit: 870 μl of 1× Click-iT reaction buffer (A), 10 μl of Alexa fluor at 500 μM (B), 15 μl of CuSO4 at 100 mM (C), 5 μl of copper protectant (D), 10 μl of 10× Click-iT buffer additive (E) and 90 μl of Milli-Q water (F). To make the reaction mix, the solutions are added in the following order: A is mixed with B, C is mixed with D, E is mixed with F, AB is mixed with CD and EF is mixed with ABCD. Cells are incubated at room temperature on a shaker at 1,000 rpm for 30 min in the dark. Cells are then spun at 17,000 rcf for 15 s, the supernatant is discarded and cells are resuspended in 800 μl of 50 mM sodium citrate and vortexed for 5 s. Cells are spun at 17,000 rcf for 15 s, the supernatant is discarded, cells are resuspended in 800 μl of 50 mM sodium citrate +1:10,000 Nuclear-ID Blue and vortexed for 5 s. Cells are spun at 17,000 rcf for 15 s, the supernatant is discarded and cells are resuspended in 800 μl of 50 μl sodium citrate. Cells are spun at 17,000 rcf for 1 s, the supernatant is discarded, cell are resuspended in 500 μl of 50 mM sodium citrate and stored at 4°C in the dark for 1 h before imaging.

Global cellular translation assay

The protocol for the global translation assay is the same as the global transcription assay, except the cells do not have the *hENT1* and *hsvTK* genes, and cells are incubated with 10 μM HPG for 5 min (4 μl from a 50 mM stock solution in Milli-Q water) instead of EU.

Flow cytometry

Before running on the flow cytometer (BD LSRFortessa; excitation laser 488 nm, long-pass filter 505 nm, bandpass filter 530/30 nm), samples are vortexed for 30 s, sonicated for 30 s (using a JSP Digital Ultrasonic Cleaner) and vortexed again for 30 s. The data are acquired using the BD FACSDiva (version 8.0.1) software. Single cells are gated based on their SSCA and FSCA profiles.

Microscopy

All bright-field and fluorescence microscopy is performed using a Nikon Eclipse Ti2 inverted microscope equipped with Nikon Perfect Focus System, Okolab environmental chamber and a Photometrics Prime Scientific CMOS camera. The microscope is controlled using the Micro-Manager v2.0 software. Fluorescence excitation is performed with a Lumencor Spectra X light engine fitted with the following excitation filters; 395/25 nm for imaging Nuclear-ID Blue; 470/24 nm for imaging EFGP and Alexa Fluor 488; 575/25 nm for imaging mCherry; and 640/30 nm for imaging Alexa Fluor 647. The emission filters used are the following: Semrock Brightline 438/24 nm for imaging Nuclear-ID Blue, Chroma ET525/50 m for imaging EFGP and Alexa Fluor 488; Semrock Brightline 641/75 nm for imaging mCherry; and Semrock Brightline 680/42 nm for imaging Alexa Fluor 647. The dichroic mirrors used are the following: Semrock 409/493/573/652 nm BrightLine quad-edge standard epi-fluorescence dichroic beam splitter for imaging Nuclear-ID Blue, EGFP, Alexa Fluor 488 and Alexa Fluor 647; and Chroma 59022bs dichroic beam splitter for imaging mCherry. Images are taken using a Nikon Plan Apo 100×/1.45 Lambda oil immersion objective.

Image segmentation and quantification

The bright-field images 1 μm below the focal plane of cells have a distinct outline and are therefore used to generate whole-cell masks using Ilastik-1.3.0-OSX. The cell masks generated this way overlap well with the cells on the focal plane images.

The three fluorescence images of the focal plane and the ± 0.5 μm z-stacks are maximum projected, and all subsequent analysis is done on the maximum projected fluorescence images.

To generate the DNA masks, Ilastik-1.3.0-OSX is used on the Nuclear-ID Blue fluorescence images.

To obtain the number of nuclei per cell, the number of DNA masks within each whole-cell mask is calculated using Fiji (ImageJ version 2.1.0/1.53c).

On all images, the scale is set using the function Analyze > Set Scale of Fiji so that the distance between 15.3609 pixels corresponds to 1 μm.

To generate single-cell measurements of cell length, the Analyze > Analyze particles function of Fiji is used on the whole-cell masks to calculate for each mask; its Feret's diameter (the longest distance between any two points within a mask used as a measurement of cell length), its area and its width (define as the width of the smallest rectangle enclosing the mask). Then, the Analyze > Analyze particles function is used with the cell masks to

© 2023 The Francis Crick Institute

calculate their corresponding fluorescence measurements on the fluorescence images, comprising the total pixel intensity, mean pixel intensity, median pixel intensity and maximum pixel intensity.

The masks are indexed so that the single-cell measurements of the different channels and the measurement of the number of nuclei are attributed to their corresponding cell mask.

The data are then processed using R (version 4.1.0) and RStudio (version 1.4.1106). For the global cellular transcription and translation signals, the median total fluorescence intensity of the background sample(s) within an experiment (cells immediately fixed after the addition of EU or HPG) is calculated. Then, the total fluorescence intensity of each cell is divided by the median background total fluorescence intensity. This allows all experiments to have fluorescence values roughly on the same scale and is convenient for processing.

Next, the background is subtracted based on cell length. Cells are grouped based on their length in bins spanning 1 μm (unless stated otherwise). For each length bin, the median background total fluorescence intensity is calculated on the background samples and subtracted from each cell's total fluorescence intensity according to its length. The total fluorescence intensity of a cell normalised by the median background total fluorescence intensity, with the median total fluorescence intensity corresponding to its length then subtracted, is used as a measure of EU or HPG incorporation.

Data availability

This study includes no data deposited in external repositories.

Expanded View for this article is available online.

Acknowledgements

We thank J. Curran, J. Greenwood, E. Roberts and N. Kapadia for their comments on the manuscript and N. Kapadia for sharing his plasmid. We also thank the Flow Cytometry and the Advanced Light Microscopy facilities at the Francis Crick Institute for their help with flow cytometry and microscopy respectively. This work was supported by the Francis Crick Institute which receives its core funding from Cancer Research UK (FC01121), the Medical Research Council (FC01121) and the Wellcome Trust (FC01121). In addition, this work was supported by the Wellcome Trust Grant to PN [grant number 214183], The Lord Leonard and Lady Estelle Wolfson Foundation and Woosnam Foundation. For the purpose of Open Access, the author has applied a CC BY public copyright licence to any Author Accepted Manuscript version arising from this submission.

Author contributions

Clovis Basier: Conceptualization; data curation; formal analysis; validation; investigation; visualization; methodology; writing – original draft; project administration; writing – review and editing. **Paul Nurse:** Conceptualization; formal analysis; supervision; funding acquisition; writing – original draft; writing – review and editing.

Disclosure and competing interests statement

All authors declare that they have no conflict of interest. Paul Nurse is a member of the Advisory Editorial Board of The EMBO Journal. This has no bearing on the editorial consideration of this article for publication.

References

Berg S, Kutra D, Kroeger T, Straehle CN, Kausler BX, Haubold C, Schiegg M, Ales J, Beier T, Rudy M *et al* (2019) Ilastik: interactive machine learning for (bio)image analysis. *Nat Methods* 16: 1226–1232

Berry S, Müller M, Rai A, Pelkmans L (2022) Feedback from nuclear RNA on transcription promotes robust RNA concentration homeostasis in human cells. *Cell Syst* 13: 454–470.e15

Buttgereit F, Brandt MD (1995) A hierarchy of ATP-consuming processes in mammalian cells. *Biochem J* 312: 163–167

Cheng L, Chen J, Kong Y, Tan C, Kafri R, Björklund M (2021) Size-scaling promotes senescence-like changes in proteome and organelle content. *bioRxiv* https://doi.org/10.1101/2021.08.05.455193 [PREPRINT]

Clemente-Blanco A, Mayán-Santos M, Schneider DA, Machín F, Jarmuz A, Tschochner H, Aragón L (2009) Cdc14 inhibits transcription by RNA polymerase I during anaphase. *Nature* 458: 219–222

Coldwell MJ, Cowan JL, Vlasak M, Mead A, Willett M, Perry LS, Morley SJ (2013) Phosphorylation of eIF4GII and 4E-BP1 in response to nocodazole treatment: a reappraisal of translation initiation during mitosis. *Cell Cycle* 12: 3615–3628

Coudreuse D, Nurse P (2010) Driving the cell cycle with a minimal CDK control network. *Nature* 468: 1074–1079

Creanor J, Mitchison JM, Williams DA (1982) Patterns of protein synthesis during the cell cycle of the fission yeast *Schizosaccharomyces pombe*. *J Cell Sci* 5: 263–285

David A, Dolan BP, Hickman HD, Knowlton JJ, Clavarino G, Pierre P, Bennink JR, Yewdell JW (2012) Nuclear translation visualized by ribosome-bound nascent chain puromycylation. *J Cell Biol* 197: 45–57

Elliott SG (1983) Coordination of growth with cell division: regulation of synthesis of RNA during the cell cycle of the fission yeast *Schizosaccharomyces pombe*. *Mol Gen Genet* 192: 204–211

Elliott SG, Mclaughlin CS (1978) Rate of macromolecular synthesis through the cell cycle of the yeast *Saccharomyces cerevisiae*. *Proc Natl Acad Sci USA* 75: 4384–43886

Elliott SG, Mclaughlin CS (1979a) Synthesis and modification of proteins during the cell cycle of the yeast *Saccharomyces cerevisiae*. *J Bacteriol* 137: 1185–1190

Elliott SG, McLaughlin CS (1979b) Regulation of RNA synthesis in yeast III. *Mol Gen Genet* 169: 237–243

Fan H, Penman S (1970) Regulation of protein synthesis in mammalian cells: II. Inhibition of protein synthesis at the level of initiation during mitosis. *J Mol Biol* 50: 655–670

Feijó Delgado F, Cermak N, Hecht VC, Son S, Li Y, Knudsen SM, Olcum S, Higgins JM, Chen J, Grover WH *et al* (2013) Intracellular water exchange for measuring the dry mass, water mass and changes in chemical composition of living cells. *PLoS One* 8: e67590

Fraser RSS, Carter BLA (1976) Synthesis of polyadenylated messenger RNA during the cell cycle of *Saccharomyces cerevisiae*. *J Mol Biol* 104: 223–242

Fraser RSS, Moreno F (1976) Rates of synthesis of polyadenylated messenger RNA and ribosomal RNA during the cell cycle of *Schizosaccharomyces pombe*. *J Cell Sci* 21: 497–521

Fraser RSS, Nurse P (1978) Novel cell cycle control of RNA synthesis in yeast. *Nature* 271: 726–730

Fraser RSS, Nurse P (1979) Altered patterns of ribonucleic acid synthesis during the cell cycle: a mechanism compensating for variation in gene concentration. *J Cell Sci* 40: 25–40

Gébrane-Younès J, Fomproix N, Hernandez-Verdun D (1997) When rDNA transcription is arrested during mitosis, UBF is still associated with non-condensed rDNA. *J Cell Sci* 110: 2429–2440

Hynes NE, Phillips SL (1976) Rate of synthesis of polyadenylate-containing ribonucleic acid during the yeast cell cycle. *J Bacteriol* 128: 502–505

 © 2023 The Francis Crick Institute

Jao CY, Salic A (2008) Exploring RNA transcription and turnover *in vivo* by using click chemistry. *Proc Natl Acad Sci USA* 105: 15779 – 15784

Konrad CG (1963) Protein synthesis and RNA synthesis during mitosis in animal cells. *J Cell Biol* 19: 266 – 277

Lanz MC, Zatulovskiy E, Swaffer MP, Zhang L, Ilerten I, Zhang S, Shin You D, Marinov GK, McAlpine P, Elias JE *et al* (2021) Increasing cell size remodels the proteome and promotes senescence. *bioRxiv* https://doi.org/10.1101/2021.07.29.454227 [PREPRINT]

Lengefeld J, Cheng CW, Maretich P, Blair M, Hagen H, McReynolds MR, Sullivan E, Majors K, Roberts C, Kang JH *et al* (2021) Cell size is a determinant of stem cell potential during aging. *Sci Adv* 7: 1 – 17

Marguerat S, Bähler J (2012) Coordinating genome expression with cell size. *Trends Genet* 28: 560 – 565

Martín-Castellanos C, Blanco MA, de Prada JM, Moreno S (2000) The puc1 cyclin regulates the G1 phase of the fission yeast cell cycle in response to cell size. *Mol Biol Cell* 11: 543 – 554

Meister P, Poidevin M, Francesconi S, Tratner I, Zarzov P, Baldacci G (2003) Nuclear factories for signalling and repairing DNA double strand breaks in living fission yeast. *Nucleic Acids Res* 31: 5064 – 5073

Meister P, Taddei A, Ponti A, Baldacci G, Gasser S (2007) Replication foci dynamics: replication patterns are modulated by S-phase checkpoint kinases in fission yeast. *EMBO J* 26: 1315 – 1326

Metzl-Raz E, Kafri M, Yaakov G, Soifer I, Gurvich Y, Barkai N (2017) Principles of cellular resource allocation revealed by condition-dependent proteome profiling. *Elife* 6: e28034

Miettinen TP, Kang JH, Yang LF, Manalis SR (2019) Mammalian cell growth dynamics in mitosis. *Elife* 8: 1 – 29

Mitchison JM (1957) The growth of single cells. I. *Schizosaccharomyces pombe*. *Exp Cell Res* 13: 244 – 262

Moreno S, Nurse P (1994) Regulation of progression through the G1 phase of the cell cycle by the rum1^{+} gene. *Nature* 367: 236 – 242

Neurohr GE, Terry RL, Lengefeld J, Bonney M, Brittingham GP, Moretto F, Miettinen TP, Vaites LP, Soares LM, Paulo JA *et al* (2019) Excessive cell growth causes cytoplasm dilution and contributes to senescence. *Cell* 176: 1083 – 1097

Nurse P, Thuriaux P, Nasmyth K (1976) Genetic control of the cell division cycle in the fission yeast *Schizosaccharomyces pombe*. *Mol Gen Genet* 146: 167 – 178

Padovan-Merhar O, Nair GP, Biaesch AG, Mayer A, Scarfone S, Foley SW, Wu AR, Churchman LS, Singh A, Raj A (2015) Single mammalian cells compensate for differences in cellular volume and DNA copy number through independent global transcriptional mechanisms. *Mol Cell* 58: 339 – 352

Patterson JO, Basu S, Rees P, Nurse P (2021) Cdk control pathways integrate cell size and ploidy information to control cell division. *Elife* 10: e64592

Prescott DM, Bender MA (1962) Synthesis of RNA and protein during mitosis in mammalian tissue culture cells. *Exp Cell Res* 26: 260 – 268

Qin X, Sarnow P (2004) Preferential translation of internal ribosome entry site-containing mRNAs during the mitotic cycle in mammalian cells. *J Biol Chem* 279: 13721 – 13728

Reid DW, Nicchitta CV (2012) The enduring enigma of nuclear translation. *J Cell Biol* 197: 7 – 9

Schmidt EE, Schibler U (1995) Cell size regulation, a mechanism that controls cellular RNA accumulation: Consequences on regulation of the ubiquitous transcription factors Oct1 and NF-Y, and the liver-enriched transcription factor DBP. *J Cell Biol* 128: 467 – 483

Scott M, Gunderson CW, Mateescu E, Zhang Z, Hwa T (2010) Interdependence of cell growth and gene expression: origins and consequences. *Science* 330: 1099 – 1102

Shuda M, Velásquez C, Cheng E, Cordek DG, Kwun HJ, Chang Y, Moore PS (2015) CDK1 substitutes for mTOR kinase to activate mitotic cap-dependent protein translation. *Proc Natl Acad Sci USA* 112: 5875 – 5882

Sivakumar S, Porter-Goff M, Patel PK, Benoit K, Rhind N (2004) *In vivo* labeling of fission yeast DNA with thymidine and thymidine analogs. *Methods* 33: 213 – 219

Stonyte V, Boye E, Grallert B (2018) Regulation of global translation during the cell cycle. *J Cell Sci* 131: jcs220327

Sun XM, Bowman A, Priestman M, Bertaux F, Martinez-Segura A, Tang W, Whilding C, Dormann D, Shahrezaei V, Marguerat S (2020) Size-dependent increase in RNA polymerase II initiation rates mediates gene expression scaling with cell size. *Curr Biol* 30: 1217 – 1230

Swaffer MP, Jones AW, Flynn HR, Snijders AP, Nurse P (2016) CDK substrate phosphorylation and ordering the cell cycle. *Cell* 167: 1750 – 1761.e16

Swaffer MP, Marinov GK, Zheng H, Jones AW, Greenwood J, Kundaje A, Snijders AP, Greenleaf WJ, Reyes-Lamothe R, Skotheim JM (2021) RNA polymerase II dynamics and mRNA stability feedback determine mRNA scaling with cell size. *bioRxiv* https://doi.org/10.1101/2021.09.20.461005 [PREPRINT]

Tanenbaum ME, Stern-Ginossar N, Weissman JS, Vale RD (2015) Regulation of mRNA translation during mitosis. *Elife* 4: e07957

Wain WH, Staatz WD (1973) Rates of synthesis of ribosomal protein and total ribonucleic acid through the cell cycle of the fission yeast *Schizosaccharomyces pombe*. *Exp Cell Res* 81: 269 – 278

Wansink DG, Schul W, van der Kraan I, van Steensel B, van Driel R, De L, Slater JEC (1993) Fluorescent labeling of nascent RNA reveals transcription by RNA polymerase II in domains scattered throughout the nucleus. *J Cell Biol* 122: 283 – 293

Zheng L, Schwartz C, Magidson V, Khodjakov A, Oliferenko S (2007) The spindle pole bodies facilitate nuclear envelope division during closed mitosis in fission yeast. *PLoS Biol* 5: 1530 – 1542

Zhurinsky J, Leonhard K, Watt S, Marguerat S, Bähler J, Nurse P (2010) A coordinated global control over cellular transcription. *Curr Biol* 20: 2010 – 2015

License: This is an open access article under the terms of the Creative Commons Attribution License, which permits use, distribution and reproduction in any medium, provided the original work is properly cited.

© 2023 The Francis Crick Institute

Expanded View Figures

Figure EV1. The assays reliably measure RNA and protein production.

A Same as Fig 1B with time 0 included. The scale bar represents 5 μm.
B Growth curves of PN1 in EMM with or without 10 μM HPG at 25°C as measured by the change in optical density at 595 nm (OD595) over time. The doubling time of each condition indicated in the legend is calculated using the slope of the OLS linear regression fitted on the data after a natural logarithmic transformation.
C HPG signal shown in Fig 1C before normalisation and background removal.
D Wild-type cells (PN1) were incubated for 30 min with 10 μM HPG, then assayed for global translation, treated with 0.05 mg/ml proteinase K at 55°C for 4 h and fluorescence was measured using flow cytometry. The 0.05, 0.25, 0.5, 0.75 and 0.95 population quantiles of at least 200,000 cells are shown.
E Wild-type cells (PN1) were spun down and resuspended in EMM (control) or EMM + 10 mg/ml cycloheximide (t = 0), then assayed for global translation at different times using flow cytometry. Population medians of at least 200,000 cells are shown.
F Cells expressing *hENT1* (PN6002), *hsvTK* (PN6003) or both (PN10597) were pulsed with 10 μM EU for 30 min and assayed for global transcription using flow cytometry. The 0.05, 0.25, 0.5, 0.75 and 0.95 population quantiles of at least 200,000 cells are shown.
G Same as Fig 1E with time 0 included. The scale bar represents 5 μm.
H Same as (B) for PN10597 grown with or without 20 μM EU.
I EU signal shown in Fig 1F before normalisation and background removal.
J Cells expressing *hENT1* and *hsvTK* (PN10597) were pulsed with 10 μM EU and labelled with Alexa Fluor 488 azide, then treated with 0.1 mg/ml RNase A at 37°C for 16 h, and the fluorescence signal was assessed using flow cytometry. The 0.05, 0.25, 0.5, 0.75 and 0.95 population quantiles of at least 200,000 cells are shown.
K Cells expressing *hENT1* and *hsvTK* (PN10597) were pulsed with EU plus DMSO or EU plus 300 μg/ml 1,10-phenanthroline, and global transcription was assayed at different time intervals using flow cytometry. Population medians of at least 200,000 cells are shown.

© 2023 The Francis Crick Institute

© 2023 The Francis Crick Institute

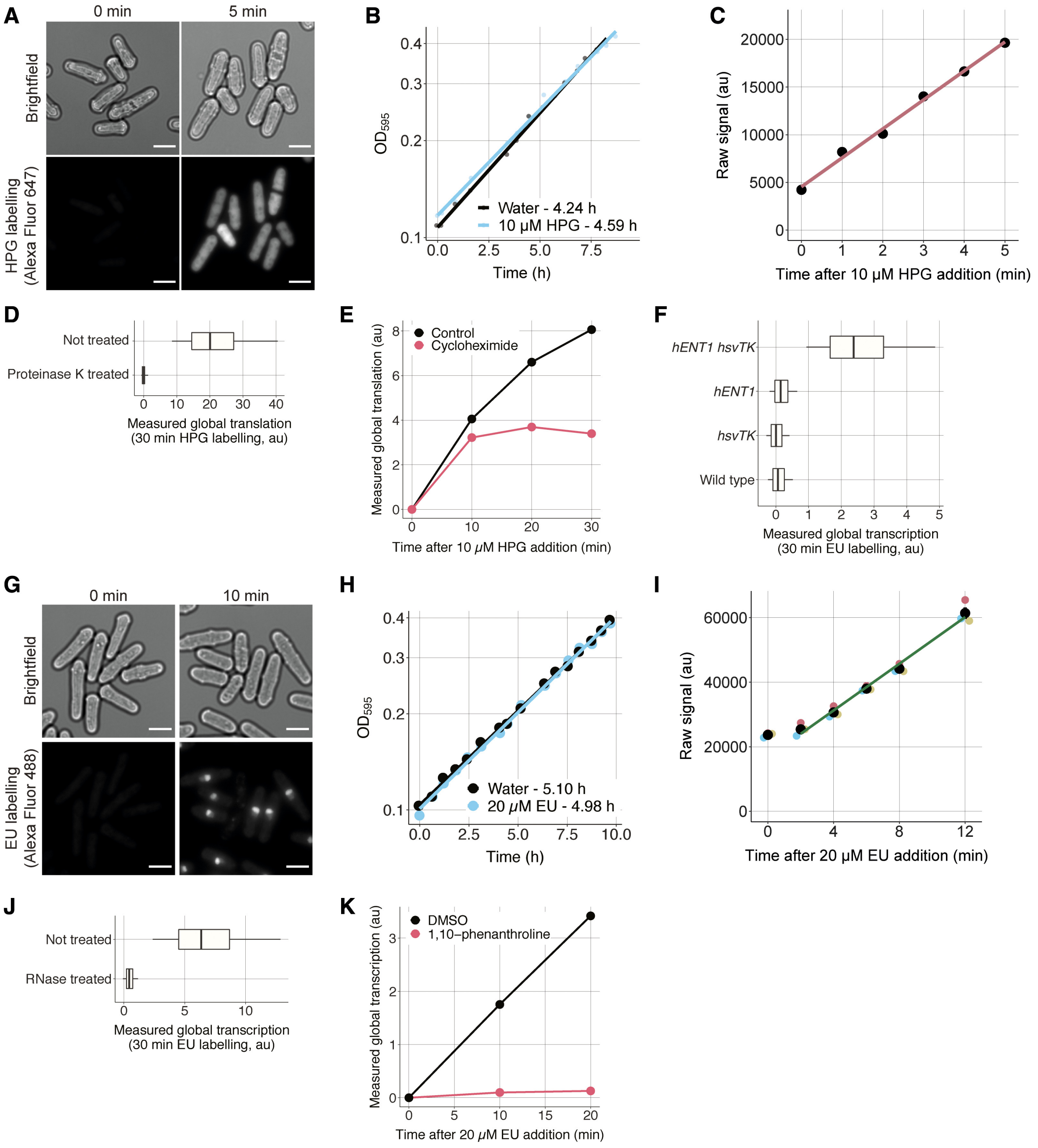
A
0 min
5 min
Brightfield
HPG labelling (Alexa Fluor 647)
B
OD595
Time (h)
Water - 4.24 h
10 μM HPG - 4.59 h
C
Raw signal (au)
Time after 10 μM HPG addition (min)
D
Not treated
Proteinase K treated
Measured global translation (30 min HPG labelling, au)
E
Control
Cycloheximide
Measured global translation (au)
Time after 10 μM HPG addition (min)
F
hENT1 hsvTK
hENT1
hsvTK
Wild type
Measured global transcription (30 min EU labelling, au)
G
0 min
10 min
Brightfield
EU labelling (Alexa Fluor 488)
H
OD595
Time (h)
Water - 5.10 h
20 μM EU - 4.98 h
I
Raw signal (au)
Time after 20 μM EU addition (min)
J
Not treated
RNase treated
Measured global transcription (30 min EU labelling, au)
K
DMSO
1,10−phenanthroline
Measured global transcription (au)
Time after 20 μM EU addition (min)

Figure EV1.

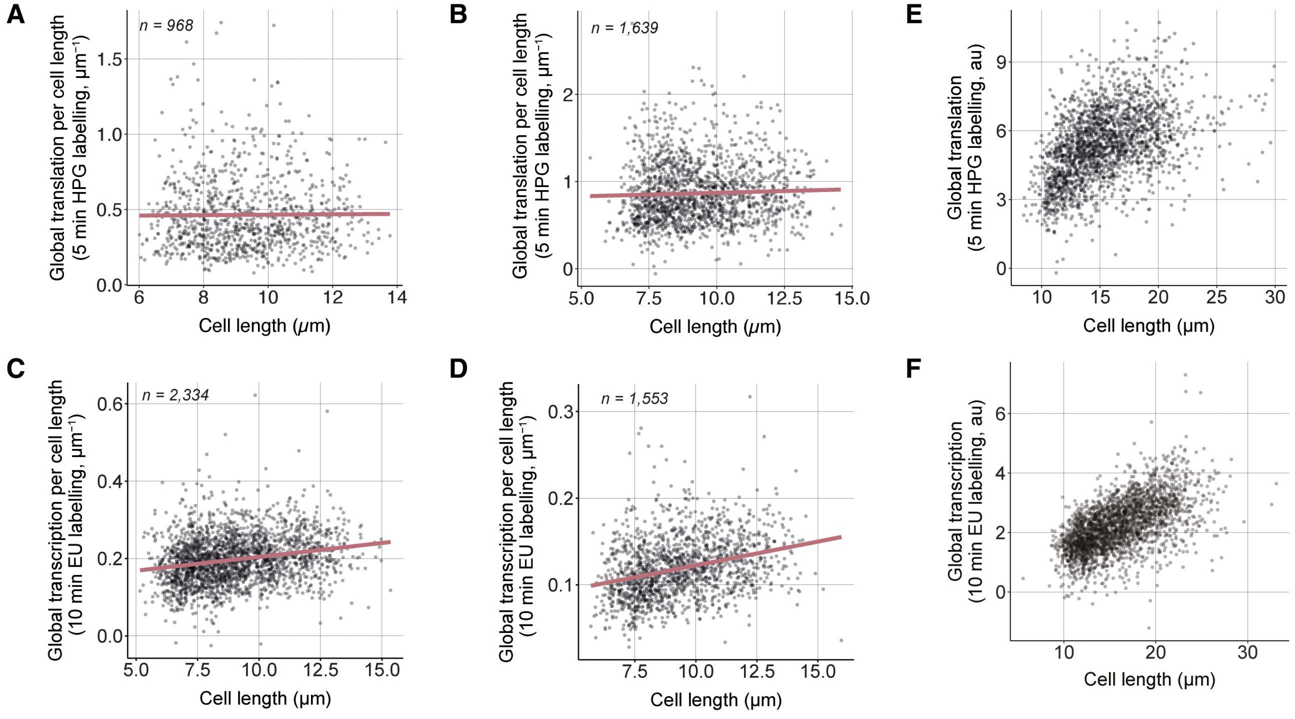

Figure EV2. Global transcription per size, but not global translation per size, somewhat increases with size.

A, B Experimental replicates of Fig 2C.

C, D Experimental replicates of Fig 2F.

E Global cellular translation of *cdc25-22* (PN143) single cells.

F Global cellular transcription of *hENT1 hsvTK cdc25-22* (PN5998) single cells.

 © 2023 The Francis Crick Institute

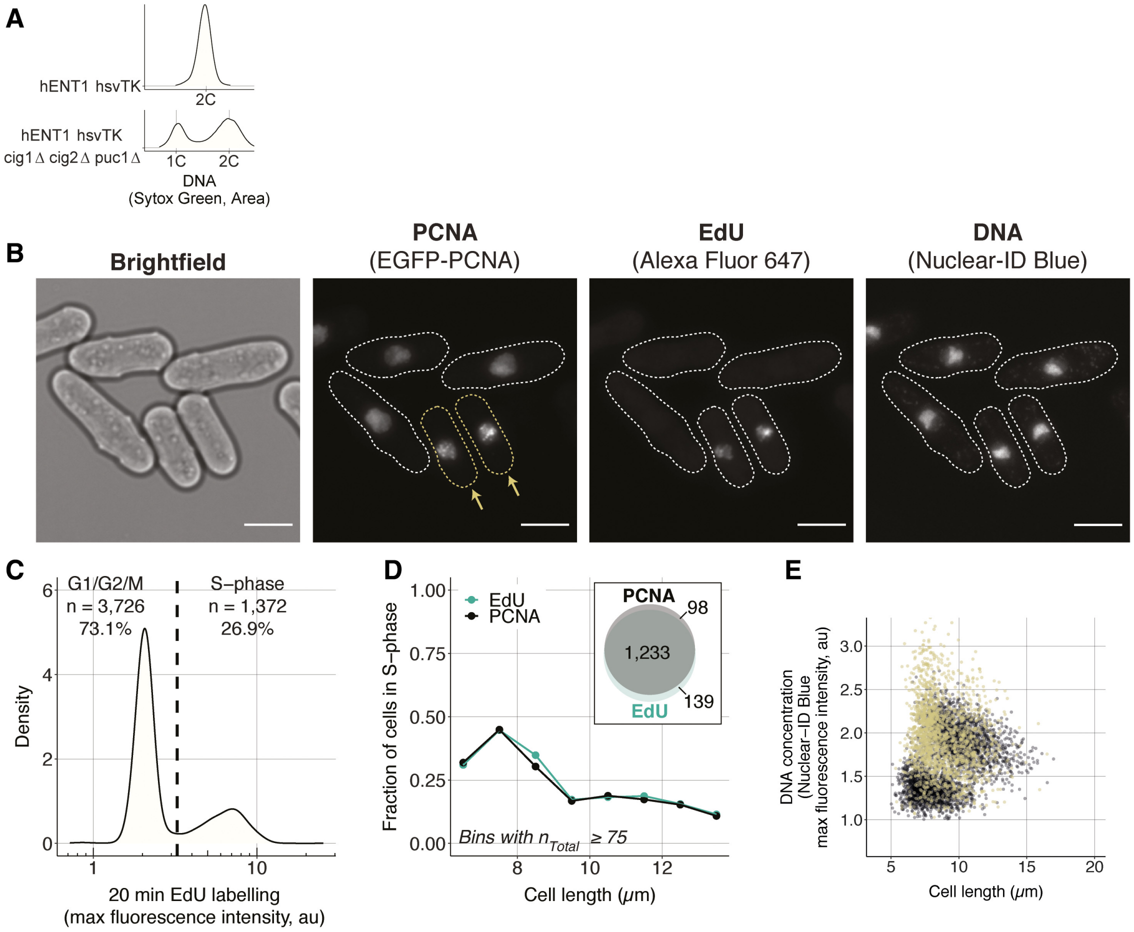

Figure EV3. The EGFP-PCNA marker allows for the accurate identification of cells in S-phase.

A Distribution of the amount of DNA in single cells in asynchronous populations, measured using the total fluorescence signal of Sytox Green by flow cytometry. For both populations, more than 200,000 cells were measured. Note that the 2C peak of the CCPΔ population (PN5999) is shifted to the right because the cells are longer and therefore have more mitochondrial DNA than the non-delete strain (PN10597).

B *hENT1 hsvTK EGFP-pcn1 CCPΔ* cells (PN6000) were pulsed with 200 μM EdU for 20 min and EdU incorporated in replicated DNA was fluorescently labelled using the same staining procedure used in the global transcription assay. Cells with visible foci in the PCNA channel are highlighted and marked with yellow arrows. The dotted white lines in the PCNA, EdU and DNA channels delimit the cell masks generated from the bright-field image. The scale bar represents 5 μm.

C Distribution of maximum fluorescence intensity of cells labelled with EdU. The dashed line represents the threshold (3.25 au) above which cells are considered in S-phase.

D The fraction of cells in S-phase per cell length is computed using the EdU signal shown in (C), or the presence of EGFP-PCNA foci determined by eye. The inset shows the overlap in cell numbers between the two methods of identifying S-phase cells.

E Same as Fig 3H with the S-phase population added in yellow.

© 2023 The Francis Crick Institute

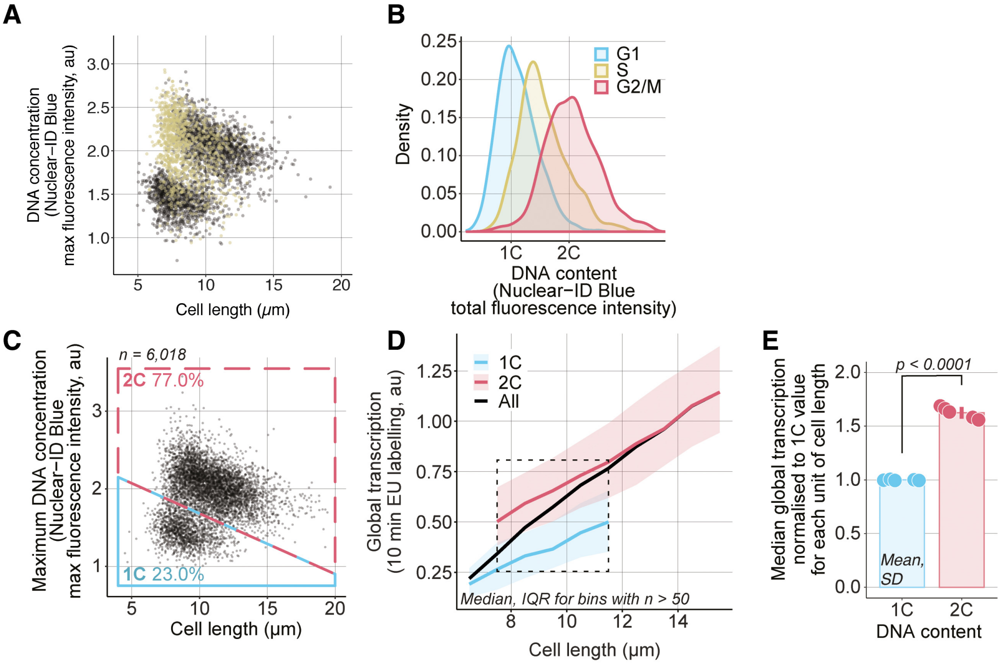

Figure EV4. A strain without the EGFP-PCNA marker also shows an increase in global transcription with cell length and from G1 to G2.

A Same as Fig 4B with the S-phase population added in yellow.

B Distribution of total DNA content of the cell populations categorised in Fig 4A and B as measured per total Nuclear-ID Blue fluorescence intensity per cell.

C *hENT1 hsvTK CCP*Δ (PN5999) cells were assayed for global transcription. The DNA concentration, measured as the maximum fluorescence intensity of the Nuclear-ID Blue stain in a cell, and cell length are used to categorise cells as having either 1C (blue box) or 2C DNA (red box). The percentage of cells in each box is shown. Black dots are single-cell measurements.

D Cells are grouped in bins of 1 µm. Medians (solid lines) and interquartile ranges (shaded areas) are shown for 1C (blue) or 2C DNA (red) populations. The dashed line box marks the length bins which have both a 1C and 2C median global transcription values.

E For each of the five length bins boxed in (B), both 1C and 2C medians are normalised to their respective global transcription 1C value. The normalised values are represented as dots, each dot corresponding to one of the five length bins, and the mean and SD of the normalised values are shown for each DNA content. The dots represent the median global transcription measurements per length bin boxed in (B). For each cell cycle stage, the normalised values (dots) are in the same order (left to right) as their corresponding length bins in (B). The *P*-value is calculated using Welch's unequal variances *t*-test.

 © 2023 The Francis Crick Institute
