# Supplementary material for: The cell cycle and cell size influence the rates of global cellular translation and transcription in fission yeast
Source: EMBO J. 2023 Mar 23;42(9):e113333. doi: 10.15252/embj.2022113333 (PMC10152140; doi:10.15252/embj.2022113333)
Supplement: Supplementary file 3 [file EMBJ-42-e113333-s001.pdf]

THE EMBO JOURNAL

The cell cycle and cell size influence the rates of global cellular translation and transcription in fission yeast

Clovis Basier and Paul Nurse
DOI: 10.15252/embj.2022113333

Corresponding author(s): Clovis Basier (clovis.basier@crick.ac.uk)

Review Timeline:

Transfer from Review Commons:	19th Dec 22
Editorial Decision:	6th Feb 23
Revision Received:	28th Feb 23
Editorial Decision:	6th Mar 23
Revision Received:	7th Mar 23
Accepted:	8th Mar 23

Review COMMONS

Editor: Hartmut Vodermaier

Transaction Report: This manuscript was transferred to The EMBO JOURNAL following peer review at Review Commons.

Review #1

1. Evidence, reproducibility and clarity:

Evidence, reproducibility and clarity (Required)

Basier and Nurse revisit the fundamental question of how the rates of RNA and protein synthesis scale with cell size. The strong null hypothesis is that synthesis scales linearly with cell size: cells that are twice as big should make stuff twice as fast. This hypothesis has been tested many times, in many systems, using many approaches over the past century and, in general, the null hypothesis has been sustained. However, there have been many examples of evidence for more complicated synthetic patterns. Whether these results indicate that biosynthesis rates vary across the cell cycle, or in response to other factors, in addition to increasing with cell size, or whether observed deviations from the predictions of the null hypothesis has been due to artifacts of cell synchronization and labeling, is thus an open, interesting and, because biosynthesis rates have critical implications in cellular function and metabolic robustness, important question.

The authors address the question in fission yeast using metabolic pulse labeling with a ribonucleoside or amino acid analog in asynchronous cells and single cell analysis to directly compare incorporation levels with cell size and cell cycle stage. The experiments are well designed, well executed and well controlled. Furthermore, the data is well presented and appropriately interpreted. In particular, the presentation of the size-v.-label data in Figures 2A and D, with the averages and variances in 2B and E and the normalized data in 2C and F are easy understand and interpret. It is thus notable that the size-v.-label data for the longer (cdc22-22) cells is omitted in favor of just the average (2H,J) and normalized (2I,K) data. This size-v.-label data should be added to Figure 2. The authors should also explicitly state how they chose 15 µm as the inflection point in 2H; 16-17 µm seems like it would give a horizontal plateau, which would better fit their saturation explanation.

The authors measure DNA content with a DNA-binding dye, the signal from which should linearly scale with DNA content. However, instead of reporting and analyzing total signal from the DNA-binding dye (or better yet, total signal in the nucleus, which they could do, having segmented the nucleus in their images), they report max signal. Using max signal is complicated because, as cells and thus nuclei increase in size the concentration of DNA and thus the max (but not total) DNA-binding-dye signal in in the nucleus decreases, requiring two-dimensional dye/size analysis (such as shown in Figure 3B) to distinguish G1 and G2 cells. The authors should use the more straight

forward measure of total nuclear DNA-binding dye signal, or explicitly explain why they can't or prefer not to do so.

The authors should state in figure legends the strain numbers used for all experiments. They should also cite the source of all the constituent parts (e.g. hENT1, hsvTK, EGFP-pcn1, and synCut3-mCherry) of their strains.

Referees cross-commenting

My colleagues make constructive points. I agree with all of them, although I am less concerned about the use of cdc2-22 and CCPΔ to alter cell length and cell cycle distribution. Although these mutations alter CDK specific activity (and thus length and distribution) and could alter specific patterns of translation, the fact that they double at normal rates makes it seem unlikely that they could be significantly changing bulk synthesis rates.

2. Significance:

Significance (Required)

As noted above, this work addresses an open, interesting and important question. Moreover it provides useful data in a specific system and a useful example of a general experimental approach to the problem. However, it does not settle the question of how biosynthesis scales with size, even in the specific case of fission yeast. In particular, it shows that protein synthesis plateaus just above normal cell size, whereas RNA synthesis scales up to twice normal cell size. This observation is striking, because there is no obvious mechanism that would (and the authors offer no suggestion of how to) explain how protein synthesis could be limited if RNA synthesis is not. Therefore, the strength of the paper is that it identifies an intriguing phenomena and its limitation is that it does not provide any testable hypotheses to explain that phenomena.

3. How much time do you estimate the authors will need to complete the suggested revisions:

Estimated time to Complete Revisions (Required)

(Decision Recommendation)

Less than 1 month

4. *Review Commons* values the work of reviewers and encourages them to get credit for their work. Select 'Yes' below to register your reviewing activity at [Web of Science Reviewer Recognition Service]() (formerly Publons); note that the content of your review will not be visible on Web of Science.

Web of Science Reviewer Recognition

Yes

Review #2

1. Evidence, reproducibility and clarity:

Evidence, reproducibility and clarity (Required)

Summary: Basier and Nurse investigate how "cell size, the amount of DNA, and cell cycle events affect the global cellular production of proteins and RNA molecules". Both transcription and translation, driving the production of biomass, have been shown to increase as a function of cell size in various systems. However, whilst cell size generally correlates with cell cycle progression there are inconsistent results in the literature if global cellular translation and transcription is affected by cell cycle state. They argue that this might be due to perturbations induced by different synchronisation methods used in the various studies.

Therefore, in this study, to avoid potential perturbation from synchronisation methods, they developed a system that allows to assay unperturbed exponentially growing populations of fission yeast cells. The assay is based on single-(fixed)cell measurements of cell size, cell cycle stage, and the levels of global cellular translation and transcription. This allows them to correlate cell cycle state, cell size and global cellular translation and transcription levels at the single cell level under unperturbed conditions.

Their results show that translation and transcription steadily increase with cell size, but that the rate of translation, but not transcription, becomes rapidly restricted when

cells become larger than wild type dividing cells. This suggests that it is unlikely that the synthesis of RNA is the limiting factor for translation rate in large cells. In addition, their data indicates that translation scales with size, but that the rate increases faster at late S-phase/early G2 and even faster in early in mitosis before decreasing in mitosis and return to interphase. Transcription, on the other hand, increases as a combination of size and the amount of DNA. Overall, this suggests that cell cycle control affects global cellular translation and transcription, which is in line with some studies, but not others. As far as I can tell the assays and data analysis are robust and the data supports the general conclusions.

Major comments

I agree that inconsistent results published on this topic might be due to perturbations induced by different synchronisation methods used in the various studies. However, but much less emphasised in the paper, it also likely depends on the model system used. For example, in budding yeast there is strong evidence for gene expression homeostasis, i.e. gene expression increases as a function of size, independent of gene copy number. Do the authors believe this is a budding yeast specific phenomenon or is this a consequence of specific synchronisation methods used in budding yeast?

Whether growth rate increases linearly or exponentially has been the topic of decade long debates. Their data indicates that the translation rate increases faster at late S-phase/early G2 and even faster early in mitosis before decreasing in mitosis and return to interphase, 'resetting' the growth rate. This suggest an exponential, rather than linear, increase in biomass (i.e. growth rate?), but this is not explicitly pointed out. It would be good to get the authors opinion on this in the discussion.

The authors state that their approach has allowed them to determine how cellular changes are arising from progression through the cell cycle. However, they use fixed cells, rather than live cell imaging, so can't claim to have established changes during cell cycle progression, but only a correlation with cell cycle state/phase. Whilst this could be used as a proxy for progression it should be clearly stated in the abstract and elsewhere to prevent confusion. I for one, based on the abstract, thought they developed a live cell imaging strategy to look at this.

In reference to the Stonyte, et al., study, in addition to different conditions (temperature shift and isoleucine medium), why do the authors think their findings are different? Is it the lack of correlation to cell size in the Stonyte paper or something else? For example, would using different growth conditions (as in the Stonyte paper). where fission yeast cells spend more time in G1, be used instead of the CCP mutant? Can the authors exclude that the lack of G1-S/cyclin-CDKs is not at the basis of a

lower rate of translation in G1 and S phase cells? Either these experiments should be carried out or this should be discussed in more detail.

If the signal to noise signal is reduced by 20 minutes EU incubation (rather than 10 minutes) why wasn't it used in all experiments? And the conclusion that the increase in transcription is not showing any discontinuities, are they referring to the triplicates in the supplementary figure 2?

Minor comments

Lines 168-169: should be Figure 2F, S2C, S2D rather than Figure 2C, S2A, S2B.

Line 179: doubling time instead of growth rate?

Lines 184-186: There is an overall trend of slight decrease in transcription per length in cdc25-22 cells but a slight increase in wild-type cells. How does this differ to wild-type cells? Are these non-significant changes and could these be attributed to the low signal to noise ratio?

There is no cell size that is specific to S phase, it falls within the range of G1 and G2 cells. Since this strain has a variable onset of S phase, the phase durations could differ. Therefore, could time spent in each phase affect the translation rate (live cell imaging, i.e. progression, could address this, but not fix cell correlation)?

The data reflects translation/transcription in single cells at a specific cell cycle phase, not during the transition between cell cycle phases. Therefore, it would be more appropriate to only use G1, S, G2 and M rather than S/G2 transition or early G2.

In figure 4C, there is a decrease in global transcription after 13 um (black line showing all cells), which they don't see in cdc25-22 mutants. Their conclusion that global transcription is constantly increasing with cell size is based on cdc-25 cells but the experiment in CCP mutant cells shows a decrease in the median of transcription. Are there replicates for these experiments as in figure 2 and supplementary figure S2? Maybe an average trend can be plotted too? Apart from the first set of experiments (figure 2 and supplementary figure 2), they don't show replicates for other strains. Maybe they can include another graph as in figure 3D and 3K of average replicate values?

Referees cross-commenting

I believe that since the whole premise of this study is that by using unperturbed

conditions their findings are different from previously published work they should either clearly point out that this difference might be due to using mutations affecting CDK activity or carry out an experiment in media that induces a G1 population. CDK has been strongly implicated in promoting translation. Using a strain that lacks the G1 and S cyclin CDKs or compromised M-CDK is therefore likely to have an effect on translation, which could be at the basis of the increase in translation during the G2 (and S) phase of the cell cycle.

2. Significance:

Significance (Required)

As far as I can tell the assays and data analysis are robust and the data supports the general conclusions. However, whilst the cells are assessed in unperturbed conditions, they do use CDK mutants and the cdc25ts mutant to establsih gene expression during the different phases of the cell cycle, which could affect translation/transcription rates. This should either be clearly pointed out or complemented with an experiment where WT cells are grown in conditions that induces distinct G1-S-G2 populations of cells.

Overall, the work presented suggests that cell cycle control affects global cellular translation and transcription, which is in line with some studies, but not others. Whilst the study falls short of testing/establishing the (potential) mechanisms involved, these are important findings, which can be used to guide new studies into how the production of biomass is controlled as cells proceed through the cell cycle.

The cell size field, which is considerable and growing, will be interested in this work.

I have expertise in cell cycle control and genome stability, with a focus on the G1-to-S transition and cell cycle checkpoints during interphase.

3. How much time do you estimate the authors will need to complete the suggested revisions:

Estimated time to Complete Revisions (Required)

(Decision Recommendation)

Between 1 and 3 months

4. *Review Commons* values the work of reviewers and encourages them to get credit for their work. Select 'Yes' below to register your reviewing activity at [Web of Science Reviewer Recognition Service]() (formerly Publons); note that the content of your review will not be visible on Web of Science.

Web of Science Reviewer Recognition

No

Review #3

1. Evidence, reproducibility and clarity:

Evidence, reproducibility and clarity (Required)

Summary

Basier and Nurse use fission yeast as a model system to investigate how transcription and translation are coupled to cell-cycle progression. They use metabolic labeling in exponentially growing cells and analyze single cells by microscopy. They find that translation scales with size and increases at S/G2 and early mitosis while transcription increases with both size and the amount of DNA. They suggest that changes in CDK activity regulate changes in global translation rates.

Major comments:

1. The paper addresses a much-disputed question in the field. The approach makes the most of the fission-yeast model system and the experiments are beautifully performed. The conclusions are well supported by the data. The experiments are replicated adequately and the statistical analyses are appropriate.
2. The use of cdc25 and in particular the cig1Δ cig2Δ puc1Δ mutants to manipulate cell size is not without challenges when monitoring translation rates. A number of reports in different model organisms suggest that CDK activity can regulate translation. Work from the Nurse lab identified translation factors as CDK substrates

(Swaffer et al, 2016), RNApolIII activity and thus tRNA levels are regulated in the cell cycle by CDK in budding yeast (Herrera et al, 2018), phosphorylation of the ribosomal protein RPL12 by CDK1 is required for translation of at least some proteins in mitosis in human cells (Imami et al, 2018), as is phosphorylation of DENR (Clemm von Hohenberg et al, 2022). The authors also suggest that changes in CDK activity might be responsible for the observed changes in global translation rates. It is important to consider whether using mutants impinging on CDK activity might lead to under- or overestimating cell-cycle dependent translation. The authors should either discuss this issue and tune down the hypothesis that CDK activity regulates changes in global translation rates, or use another approach to address the issue. One could use a replication mutant such as cdc17 or cdc20 to alter cell size without interfering with CDK activity. These experiments would strengthen the conclusions and might support the idea that CDK activity regulates changes in global translation rates.

References

Clemm von Hohenberg K, Müller S, Schleich S, Meister M, Bohlen J, Hofmann TG, Teleman AA (2022) Cyclin B/CDK1 and Cyclin A/CDK2 phosphorylate DENR to promote mitotic protein translation and faithful cell division. Nat Commun 13: 668

Herrera MC, Chymkowitch P, Robertson JM, Eriksson J, Bøe SO, Alseth I, Enserink JM (2018) Cdk1 gates cell cycle-dependent tRNA synthesis by regulating RNA polymerase III activity. Nucleic Acids Res 46: 11698-11711

Imami K, Milek M, Bogdanow B, Yasuda T, Kastelic N, Zauber H, Ishihama Y, Landthaler M, Selbach M (2018) Phosphorylation of the Ribosomal Protein RPL12/uL11 Affects Translation during Mitosis. Mol Cell 72: 84-98 e89

Swaffer MP, Jones AW, Flynn HR, Snijders AP, Nurse P (2016) CDK Substrate Phosphorylation and Ordering the Cell Cycle. Cell 167: 1750-1761 e1716

Minor comments:

1. The figures are beautifully presented, easy to understand and the cartoons present the experimental strategies very clearly.
2. A major feature of the approach is that translation and transcription are monitored in exponentially growing cells, which are not exposed to any stress such as cell-cycle synchronization. However, one could argue that the analogues used for labeling impose some kind of stress, even if this is not very likely at the labeling times employed. A simple control experiment where the growth rates of labeled and unlabeled cells are compared would strengthen the claim that these are indeed happily

growing cells.
3. Please comment why the length of the EU labeling differs from figure to figure. In fig 2C, S2C and S2D the labeling on the y axes states 10 min, in Fig 4C it says 20 min.
4. Lines 118-119 "The pulse signal was five times the background signal." Figure S2A,B show large variation in signal intensity after 5 min labelling. It is not clear how the pulse signal was estimated to be five times the background signal.
5. In Fig S4C transcription is up by ca 60 % from G1 to G2, while in Fig 4D transcription is up by ca 25-30%, also from G1 to G2. The only difference I can see is the use of PCNA-GFP. Please comment what the reason might be.
6. Fig 1 B images of unlabeled control cells should also be shown.
7. Lines 156 "to investigate how global cellular translation and transcription are affected by cell size, and by progression through the cell cycle" should be amended. Throughout the description of data in figure 2 binucleated and septated cells were excluded from the analyses, meaning that the data only represent cells in G2. The text should make this clear.
8. Lines 241-243 "the S-phase subpopulation was found to have an intermediary global transcription value between the G1 and G2/M subpopulations of around 20-25 %." And Lines 310-313 "the rate of transcription is increased in cells undergoing S-phase by 20 % and is 35 % higher in G2 cells which have completed S-phase, indicating that DNA content is limiting the global rate of transcription." It is unclear what the percentage values refer to and which populations exactly are being compared.
9. Line 85 "Asynchronous cultures ... have not detected" rephrase; change detected to displayed or similar.
10. Line 243 Figure 4J, K should read Figure 4C, D.

Referees cross-commenting

I also agree with the comments made by the colleagues. As for the use of the cyclin and cdc25 mutants: I agree with Reviewer #1 that it is unlikely that bulk synthesis rates are conisedarably different, since these strains are going at more or lass normal rates. However, I also agree with reviewer #2 that these mutants cannot be considered as unperturbed conditions. I suspect subtle regulation and in particular cell-cycle dependent regulation might well be lost. At the very least the focus of the interpretation should be on translation/transcription as a function of size, rather than in terms of cell-cycle regulation.

2. Significance:

Significance (Required)

Basier and Nurse address a long-standing question in the cell-cycle field, namely how/whether transcription and translation are coupled to cell-cycle progression. This is technically challenging to address, and many previous studies were hampered by the necessity to synchronize the cells in the cell cycle. The approach of this study of using metabolic labeling in non-synchronized cells is not novel in itself. However, the analysis by microscopy is superior to previous flow-cytometry based strategies in that it allows the use of cell-cycle markers and thereby precise identification of cells in each cell-cycle phase. In addition, it allows accurate measurements of cell size and thus addressing questions of correlations between cell size and transcription / translation rates. A further strength of the study design is that they investigate both transcription and translation in parallel.

The authors very nicely review the existing literature and point out the likely reasons for conflicting conclusions (synchronization methods, choice of model system). The advantages of their approach, such as single-cell analyses in non-synchronized cells and the use of cell-cycle markers make their conclusions less likely to be flawed and thus represent an important advance in the field.

These findings are of interest for researchers working on the cell-cycle field and on the translation field. There have been significant technical advances in the translation field in recent years, allowing studying not only global translation but also translation of specific mRNAs. I expect that the old questions of coupling cell cycle and cell growth will be revisited also by others, exploiting these new approaches.
My field of expertise extends to the cell-cycle field and the regulation of translation and the use of fission yeast.

3. How much time do you estimate the authors will need to complete the suggested revisions:

Estimated time to Complete Revisions (Required)

(Decision Recommendation)

Between 1 and 3 months

4. *Review Commons* values the work of reviewers and encourages them to get credit for their work. Select 'Yes' below to register your reviewing activity at Web of Science Reviewer Recognition Service (formerly Publons); note that

the content of your review will not be visible on Web of Science.

Web of Science Reviewer Recognition

Yes

Review #4

1. Evidence, reproducibility and clarity:

Evidence, reproducibility and clarity (Required)

Summary

Single cell measurements (flow cytometry and imaging) from unperturbed cells are obtained to investigate scaling of transcription and translation in fission yeast. A key finding is that translation and transcription are somewhat differentially responding to changes in cell size and cell cycle. Perhaps the most central finding of this manuscript is that transcription is not a limiting factor to translation and suggests that transcription is not limiting growth (increase in biomass).

Major comments:

What I like in this manuscript is that the translation and transcription measurements have been carefully checked to reflect the initial rates before the HPG and EU signals lose their linearity. More generally, experiments have been conducted with appropriate controls, and the analysis of unperturbed cells in each cell cycle phase is likely to be highly relevant for resolving some of the controversies in the field. Most claims and the conclusions are well supported by the data.

Although it is encouraging that the results for translation match the single cell mass measurements in mammalian cells (e.g., ref 18), I would have liked to see some more discussion about the potential caveats of the performed analyses such as the low signal to noise ratio in EU incorporation and other potential technical issues, which might have confounded the results. As an example, looking at Figs 1B and E, most of the protein and RNA synthesis signal is nuclear localized. Is this due to nucleolar

staining and incorporation of the labels into nascent ribosomes? Yet the manuscript mentions that roughly half of RNA is for rRNA and for ribosomal proteins the fraction of HPG incorporation might be even lower. This statement does not sound entirely consistent with the experimental images shown in Fig 1. Please clarify.

A curious thing that has been glossed over is that the transcription and translation seem not to be completely linear but to display opposite patterns (translation slightly reducing, transcription slightly overshooting with cell size compared to a linear model). It remains possible that this could be experimental noise and a visual pattern that is not real, but it could also be relevant for growth control. For example, my interpretation from Fig. 2B is that the signal is not linear and starts to saturate around 10.5 um cell length as seen from the upper IQR. Related to this, I think it is oversimplification to force the data to appear as a discontinuous linear trend by splitting the data in 2H into two segments. Such a treatment will obviously match the data better than a single linear regression, but perhaps some nonlinear model would be actually much more accurate, unless you can point out some kind of regulatory event at the intersection of these two linear segments. In my opinion the current data looks more like a typical (logarithmic) growth curve of the cell population reaching saturation. Please comment.

The main conclusion presented in the abstract is that scaling in transcription may result from dynamic equilibrium between RNA polymerases and available DNA template. This is a bit of speculative part, which I was not too fond of. The dynamic equilibrium idea has been suggested also elsewhere (refs 47) and is not well developed in this manuscript. There is a lack of mechanistic understanding and no formal (mathematical) model to support this idea. For example, global transcription increases much less (1.3-1.4x) than expected based on the increase in DNA content from G1 to G2 (2x). Is this expected based on the dynamic equilibrium model?

I am somewhat concerned about the interpretation of the S phase data in the global transcription measurement. The quantification in Fig. 4D shows S phase being intermediate between G1 and G2. Yet, when you look at the data in Fig. 4C, the S phase median is clearly discontinuous, with higher transcription in smaller S cells. I believe this could affect the normalized data in Fig. 4D and result in the apparent increase in transcription in S phase cells. Having said that, I am not sure if this small S phase transcription is noise (low cell counts?) or a real S phase specific regulation of transcription which is not DNA content dependent. This results is one of the most central ones in this paper to differentiate between transcriptional and translational scaling. Therefore, additional data or insights would be highly appreciated.

Minor comments:

Line 61: "patterns of protein RNA". I guess this refers to patterns of protein/RNA synthesis?

Line 248: typo "Tanslation"

Line 410 and 416: Move interquartile ranges from line 416 to line 410 as this is the first occurrence of the IQR abbreviation.

Line 473: "Almost linear". This is a subjective expression, please provide some measure such as the R2 value to quantitatively evaluate linearity in this strain.

Line 547: Is there a reason to stress in this experiment that the AREA of the fluorescence signal was measured as the area indicates the total fluorescence intensity?

Fig1A: The schematic mentions peptides, shouldn't it be more accurate to use "polypeptides" or "proteins" when discussing protein synthesis?Fig 5G: Y axis scale has a typo in the word transcription

Referees cross-commenting

I also agree with the points raised by the colleagues. There will always be some technical or interpretation issues related to every experimental technique, every model system and every mutant strain used. I believe after addressing these limitations as pointed out in the reviews, most of those issues have been clarified for the readers.

2. Significance:

Significance (Required)

Basier and Nurse revisit the classic question regarding growth and cell size control by examining scaling of global translation and transcription in fission yeast.
Knowing how cells alter their transcription and translation has important consequences in cellular functions during proliferation and cellular aging and is of broad general interest. The main driver for this current work is that previous experiments both in fission yeast and other model organisms have yielded conflicting results, possibly due to different cell cycle synchronization methods. The strength of the paper is indeed in the single cell analyses of well defined yeast strains which allow accurate assessment of the cell cycle dependent changes and accurate measurements of cell size using the cell length.

Reassuringly, the single cell analyses from unperturbed yeast cells resemble those recently obtained from unperturbed growth of individual mammalian cells. The main conclusion that transcription is not limiting translation, and consequently not limiting growth of the cells, is interesting as it is not consistent with some of the prevailing ideas in the cell size field. These ideas include ploidy dependent gene expression where DNA content is thought to be limiting growth or the model for minimal gene expression which assumes RNA polymerases are limiting gene expression and growth. In this regard, this manuscript provides important insights for future thinking of how growth is controlled.

keywords: cell cycle, cell size control

3. How much time do you estimate the authors will need to complete the suggested revisions:

Estimated time to Complete Revisions (Required)

(Decision Recommendation)

Between 1 and 3 months

4. *Review Commons* values the work of reviewers and encourages them to get credit for their work. Select 'Yes' below to register your reviewing activity at Web of Science Reviewer Recognition Service (formerly Publons); note that the content of your review will not be visible on Web of Science.

Web of Science Reviewer Recognition

Yes

Full Revision

Manuscript number: RC- 2022-01718
Corresponding author(s): Clovis, Basier

[Please use this template only if the submitted manuscript should be considered by the affiliate journal as a full revision in response to the points raised by the reviewers.

If you wish to submit a preliminary revision with a revision plan, please use our "Revision Plan" template. ***It is important to use the appropriate template to clearly inform the editors of your intentions.****]*

1. General Statements [optional]

This section is optional. Insert here any general statements you wish to make about the goal of the study or about the reviews.

It is well established that cells maintain approximately constant concentrations of proteins and RNA through growth and cell reproduction. Maintaining an optimal concentration of RNA and proteins is crucial as highlighted by recent work suggesting that cytoplasmic dilution of proteins drives aging and senescence. Yet, it is still unclear how cells achieve maintenance of these stable concentrations. Particularly, how the production of RNA and protein is influenced as cells grow and progress through the various events of the cell cycle have been investigated for decades but different studies have generated varied and inconsistent results likely due to the different methods used to generate synchronous cultures.

In this work, we develop a single-cell approach using unperturbed exponentially growing cultures of the fission yeast *Schizosaccharomyces pombe* to describe the contributions of cell size and cell cycle stages to the scaling of the global production of RNA and proteins, thus avoiding the potential biases that may have affected most previous studies.

We find that translation and transcription follow different patterns of scaling. Global cellular translation increases with size and is modulated at discrete cell cycle events, at the S/G2 transition and in mitosis. Thus, we demonstrate that there is a cell cycle regulation of global translation. Global cellular transcription increases with both cell size and during S-phase, thus supporting the recent idea that RNA polymerases are associating and disassociating from DNA in a dynamic equilibrium.

Our data provides an extensive and accurate characterisation in unperturbed cells of global cellular translation and transcription through G1, S, G2, and mitosis. We show that growth, through global cellular translation and transcription, is directly impacted by progression through the cell cycle. These conclusions are important for understanding the processes underlying global cellular growth, a somewhat neglected area of study.

We note that all the reviewers agree on the importance of the question and that our results provide important insights for the cell growth and cell cycle fields. They also agree that the experiments are well designed and presented, and that our analysis is robust.

2. Point-by-point description of the revisions

This section is mandatory. Please insert a point-by-point reply describing the revisions that were already carried out and included in the transferred manuscript.

Below are the reviewer comments (in black), and our answers (in grey) detailing the revisions made to the manuscript.

Reviewer #1 (Evidence, reproducibility and clarity (Required)):

Basier and Nurse revisit the fundamental question of how the rates of RNA and protein synthesis scale with cell size. The strong null hypothesis is that synthesis scales linearly with cell size: cells that are twice as big should make stuff twice as fast. This hypothesis has been tested many times, in many systems, using many approaches over the past century and, in general, the null hypothesis has been sustained. However, there have been many examples of evidence for more complicated synthetic patterns. Whether these results indicate that biosynthesis rates vary across the cell cycle, or in response to other factors, in addition to increasing with cell size, or whether observed deviations from the predictions of the null hypothesis has been due to artifacts of cell synchronization and labeling, is thus an open, interesting and, because biosynthesis rates have critical implications in cellular function and metabolic robustness, important question.

The authors address the question in fission yeast using metabolic pulse labeling with a ribonucleoside or amino acid analog in asynchronous cells and single cell analysis to directly compare incorporation levels with cell size and cell cycle stage. The experiments are well designed, well executed and well controlled. Furthermore, the data is well presented and appropriately interpreted. In particular, the presentation of the size-v.-label data in Figures 2A and D, with the averages and variances in 2B and E and the normalized data in 2C and F are easy understand and interpret. It is thus notable that the size-v.-label data for the longer (cdc22-22) cells is omitted in favor of just the average (2H,J) and normalized (2I,K) data. This size-v.-label data should be added to Figure 2.
We added two panels to the Figure supplementary 2 showing the requested data, the size-v.-global translation (S2E) and size-v.-global transcription (S2F).

The authors should also explicitly state how they chose 15 µm as the inflection point in 2H; 16-17 µm seems like it would give a horizontal plateau, which would better fit their saturation explanation.
This comment relates to the second comment of reviewer 4, see below for the detailed answer.

The authors measure DNA content with a DNA-binding dye, the signal from which should linearly scale with DNA content. However, instead of reporting and analyzing total signal from the DNA-binding dye (or better yet, total signal in the nucleus, which they could do, having segmented the nucleus in their images), they report max signal. Using max signal is complicated because, as cells and thus nuclei increase in size the concentration of DNA and thus the max (but not total) DNA-binding-dye signal in in the nucleus decreases, requiring two-dimensional dye/size analysis (such as shown in Figure 3B) to distinguish G1 and G2 cells. The authors should use the more straight forward measure of total nuclear DNA-binding dye signal, or explicitly explain why they can't or prefer not to do so.
The total fluorescence intensity signal of the DNA-binding dye is noisy because we had to use a low concentration of the dye. This was necessary as it allows a clearer distinction between cells with a one 1C DNA content and cells with a 2C DNA content that higher concentrations did not. The maximum signal per cell-v.-cell length produces distinct populations of cells in G1, or G2/M phase (see Figure 3H, and Figure 4B), and populations identified in this way have the distributions of total fluorescence intensity expected from cells in G1 and G2 or M phase (see Figure 3I and Figure S4D). We added one extra panel to Supplementary Figure 4 showing the distributions of the total fluorescence intensity signal of the DNA-binding dye for the G1, S, and G2 or M populations (S4D) for comparison.

The authors should state in figure legends the strain numbers used for all experiments.
We have modified all the figure legends to include the strain numbers.

They should also cite the source of all the constituent parts (e.g. hENT1, hsvTK, EGFP-pcn1, and synCut3-mCherry) of their strains.
The missing reference for the source of hENT1 and hsvTK (Sivakumar et al. 2004) has been added, the references for EGFP-pcn1 (Meister et al., 2003) and synCut3-mCherry (Patterson et al., 2021) were already present.

CROSS-CONSULTATION COMMENTS
My colleagues make constructive points. I agree with all of them, although I am less concerned about the use of cdc2-22 and CCPΔ to alter cell length and cell cycle distribution. Although these mutations alter CDK specific activity (and thus length and distribution) and could alter specific patterns of translation, the fact that they double at normal rates makes it seem unlikely that they could be significantly changing bulk synthesis rates.

Reviewer #1 (Significance (Required)):

As noted above, this work addresses an open, interesting and important question. Moreover it provides useful data in a specific system and a useful example of a general experimental approach to the problem. However, it does not settle the question of how biosynthesis scales with size, even in the specific case of fission yeast. In particular, it shows that protein synthesis plateaus just above normal cell size, whereas RNA synthesis scales up to twice normal cell size. This observation is striking, because there is no obvious mechanism that would (and the authors offer no suggestion of how to) explain how protein synthesis could be limited if RNA synthesis is not. Therefore, the strength of the paper is that it identifies an intriguing phenomena and its limitation is that it does not provide any testable hypotheses to explain that phenomena.

Reviewer #2 (Evidence, reproducibility and clarity (Required)):

Summary: Basier and Nurse investigate how "cell size, the amount of DNA, and cell cycle events affect the global cellular production of proteins and RNA molecules". Both transcription and translation, driving the production of biomass, have been shown to increase as a function of cell size in various systems. However, whilst cell size generally correlates with cell cycle progression there are inconsistent results in the literature if global cellular translation and transcription is affected by cell cycle state. They argue that this might be due to perturbations induced by different synchronisation methods used in the various studies.

Therefore, in this study, to avoid potential perturbation from synchronisation methods, they developed a system that allows to assay unperturbed exponentially growing populations of fission yeast cells. The assay is based on single-(fixed)cell measurements of cell size, cell cycle stage, and the levels of global cellular translation and transcription. This allows them to correlate cell cycle state, cell size and global cellular translation and transcription levels at the single cell level under unperturbed conditions.

Their results show that translation and transcription steadily increase with cell size, but that the rate of translation, but not transcription, becomes rapidly restricted when cells become larger than wild type dividing cells. This suggests that it is unlikely that the synthesis of RNA is the limiting factor for translation rate in large cells. In addition, their data indicates that translation scales with size, but that the rate increases faster at late S-phase/early G2 and even faster in early in mitosis before decreasing in mitosis and return to interphase. Transcription, on the other hand, increases as a combination of size and the amount of DNA. Overall, this suggests that cell cycle control affects global cellular translation and transcription, which is in line with some studies, but not others. As far as I can tell the assays and data analysis are robust and the data supports the general conclusions.

Major comments
I agree that inconsistent results published on this topic might be due to perturbations induced by different synchronisation methods used in the various studies. However, but much less

emphasised in the paper, it also likely depends on the model system used. For example, in budding yeast there is strong evidence for gene expression homeostasis, i.e. gene expression increases as a function of size, independent of gene copy number. Do the authors believe this is a budding yeast specific phenomenon or is this a consequence of specific synchronisation methods used in budding yeast?
Gene expression homeostasis has been suggested for budding yeast, but in contrast recent work in budding yeast also suggests that gene expression increases with the genome copy number and therefore the gene copy number in addition to cell size (Swaffer et al., 2022 – currently on bioRxiv). The differences that have been reported might be due to perturbations such as synchronisation methods as well as differences between yeast species.

Whether growth rate increases linearly or exponentially has been the topic of decade long debates. Their data indicates that the translation rate increases faster at late S-phase/early G2 and even faster early in mitosis before decreasing in mitosis and return to interphase, 'resetting' the growth rate. This suggest an exponential, rather than linear, increase in biomass (i.e. growth rate?), but this is not explicitly pointed out. It would be good to get the authors opinion on this in the discussion.
Assuming that protein degradation remains constant throughout growth, the increase of translation with cell size suggests that the growth rate increases as cells grow in size, possibly exponentially. In addition, our data showing that the translation rate increases from G1 to G2 for the same cell size, suggests that for cells of a given size the growth rate is faster in G2 than in G1. Thus, growth could be basically exponential but the speed of increase accelerates at the transition between S and G2, and early in mitosis, slowing down later in mitosis. We added the following sentence to the discussion section "Global transcription and translation increase with cell size possibly exponentially, but the changes in global translation during transitions through cell cycle stages suggest that the speed of growth is modulated by cell cycle progression, increasing between S and G2, and early in mitosis, and slowing down later in mitosis.".

The authors state that their approach has allowed them to determine how cellular changes are arising from progression through the cell cycle. However, they use fixed cells, rather than live cell imaging, so can't claim to have established changes during cell cycle progression, but only a correlation with cell cycle state/phase. Whilst this could be used as a proxy for progression it should be clearly stated in the abstract and elsewhere to prevent confusion. I for one, based on the abstract, thought they developed a live cell imaging strategy to look at this.
We have modified the abstract to reflect the fact that the cells were fixed in our assays (line 36).

In reference to the Stonyte, et al., study, in addition to different conditions (temperature shift and isoleucine medium), why do the authors think their findings are different? Is it the lack of correlation to cell size in the Stonyte paper or something else? For example, would using different growth conditions (as in the Stonyte paper). where fission yeast cells spend more time

in G1, be used instead of the CCP mutant? Can the authors exclude that the lack of G1-S/cyclin-CDKs is not at the basis of a lower rate of translation in G1 and S phase cells? Either these experiments should be carried out or this should be discussed in more detail.
In the study carried out by Stonyte et al., the relative translation rate per cell (a measurement related to our measurement of translation normalised per unit of length) of wild type fission yeast cells grown asynchronously in isoleucine minimal medium is constant between the G1 and the S phase cell populations, and is higher in the G2 population compared to the S phase population (Figure 2D of Stonyte et al., 2018). This is consistent with the lack of increase that we observe for a given cell size from G1 to G2, and the increase we observe from S to G2 in Figure 3K. In the same figure, Stonyte at al., find no difference between the G2 and the M-G1 populations but are not able to distinguish cells at different stages of mitosis or in early G1. Our study suggests that translation increases early in mitosis before decreasing after anaphase A, thus in the Stonyte et al study, pooling all stages of mitosis and early G1 cells might mask the dynamics of what is happening during mitosis. The lack of G1-S/cyclin-CDK could be the basis for the lower rate of translation in G1 and S-phase. We discuss this further in a reply to the first question of the significance part of reviewer 2 and have added a section to the discussion of the paper (see below for details).

If the signal to noise signal is reduced by 20 minutes EU incubation (rather than 10 minutes) why wasn't it used in all experiments?
To measure RNA production as closed as possible to the instantaneous rate of RNA synthesis, we sought to use the shortest pulse possible. We did this because the half-lives of some RNA species are short, in particular, the half-life of the pool of mRNA has been reported to be around 13.1 minutes in budding yeast (Chan et al., 2017). In longer pulses, some RNA molecules that have been synthesised after addition of EU will therefore have been degraded before cells are fixed, producing a measurement that underestimates the rate of RNA synthesis. We chose to incubate cells for 10 minutes as we estimated it to be the shortest time generating a signal to noise ration above 1 (Figure 1F). The one exception to this was with the pulsing of the CCP∆ EGFP-pcn1 hENT1 hsvTK mutant cells which incorporates less EU during the same time frame so we incubated this strain for 20 minutes to generate enough signal to be quantifiable (see line 237, "we assayed CCP∆ EGFP-pcn1 hENT1 hsvTK cells for global transcription using a 20-minute EU incubation to compensate for their lower signal production").

And the conclusion that the increase in transcription is not showing any discontinuities, are they referring to the triplicates in the supplementary figure 2?
We think there might be a misunderstanding. We conclude that the increase in transcription shows no discontinuity because the median transcription increases steadily with cell length in Figure 2E. We have added "since global transcription increases smoothly with cell length (Figure 2E)" to clarify the text.

Minor comments
Lines 168-169: should be Figure 2F, S2C, S2D rather than Figure 2C, S2A, S2B.
The figure numbers have been corrected in the manuscript.

Line 179: doubling time instead of growth rate?
The mention of "growth rate" has been changed to "doubling time" in the manuscript.

Lines 184-186: There is an overall trend of slight decrease in transcription per length in cdc25-22 cells but a slight increase in wild-type cells. How does this differ to wild-type cells? Are these non-significant changes and could these be attributed to the low signal to noise ratio?
These changes may be due to the low signal to noise ratio in the cdc25-22 transcription assay. We have added "The decrease with cell length in transcription that we observe in the cdc25-22 hENT1 hsvTK (Figure 2K) cells but not in the hENT1 hsvTK cells (Figure 2F) may be due to the low signal to noise ratio".

There is no cell size that is specific to S phase, it falls within the range of G1 and G2 cells. Since this strain has a variable onset of S phase, the phase durations could differ. Therefore, could time spent in each phase affect the translation rate (live cell imaging, i.e. progression, could address this, but not fix cell correlation)?
It is possible that the phase duration of G1 and G2 could differ from one cell to another. There is no evidence that the length of S-phase varies in these cells. It would be interesting to measure how the phase length influences translation, but our techniques do not allow for the measurement of global translation in living cells.

The data reflects translation/transcription in single cells at a specific cell cycle phase, not during the transition between cell cycle phases. Therefore, it would be more appropriate to only use G1, S, G2 and M rather than S/G2 transition or early G2.
Our data represents cells at fixed cell cycle phases and we do not monitor the transition themselves directly. However, the discontinuity in signal for cells of the same size in consecutive stages of the cell cycle (for instance the discontinuity in translation between S and G2 cells of the same size in Figure 3J) is indicative that the transition between the two cell cycle phases is a consequence of a rate change.

In figure 4C, there is a decrease in global transcription after 13 um (black line showing all cells), which they don't see in cdc25-22 mutants. Their conclusion that global transcription is constantly increasing with cell size is based on cdc-25 cells but the experiment in CCP mutant cells shows a decrease in the median of transcription. Are there replicates for these experiments as in figure 2 and supplementary figure S2? Maybe an average trend can be plotted too? Apart from the first set of experiments (figure 2 and supplementary figure 2), they don't show replicates for other strains. Maybe they can include another graph as in figure 3D and 3K of average replicate values?

Review COMMONS

The apparent decrease in transcription on Figure 4C in long cells is seen in only one length bin (13.5 µm), which has a smaller number of cells compared to the ones directly before (89 cells, compared to 216 cells for the 12.5 µm bin and 316 cells for the 11.5 µm bin). This might have resulted in a higher variability in the measurement of the population median. We do not see the same decrease at 13.5 µm in the wild type (Fig 5G), the cdc25-22 mutant (Fig 2J), or the CCP∆ strain (Fig S4B) so on balance we favour the interpretation that the decrease observed in the longer length bin of Figure 3J is due to variability caused by the lower number of cells in that bin.

CROSS-CONSULTATION COMMENTS
I believe that since the whole premise of this study is that by using unperturbed conditions their findings are different from previously published work they should either clearly point out that this difference might be due to using mutations affecting CDK activity or carry out an experiment in media that induces a G1 population. CDK has been strongly implicated in promoting translation. Using a strain that lacks the G1 and S cyclin CDKs or compromised M-CDK is therefore likely to have an effect on translation, which could be at the basis of the increase in translation during the G2 (and S) phase of the cell cycle.
This is addressed in the next comment.

Reviewer #2 (Significance (Required)):

As far as I can tell the assays and data analysis are robust and the data supports the general conclusions. However, whilst the cells are assessed in unperturbed conditions, they do use CDK mutants and the cdc25ts mutant to establsih gene expression during the different phases of the cell cycle, which could affect translation/transcription rates. This should either be clearly pointed out or complemented with an experiment where WT cells are grown in conditions that induces distinct G1-S-G2 populations of cells.
The cell cycle stage and CDK activity are intrinsically linked. CDK activity defines the cell cycle stage so that an increase in CDK activity through the cell cycle is responsible for cells progressing through G1, S, G2, and mitosis (Coudreuse and Nurse, 2010, Swaffer et al., 2016). Nutritional conditions that induce a G1 also rely on repression of CDK activity through increased production of the Rum1 inhibitor (Rubio et al., 2018) to generate a G1 population. Therefore, uncoupling CDK activity from the cell cycle would not be possible in an unperturbed cell population. We have added the following paragraph to the discussion to address the comment "The cell cycle stage of a cell and the activity of its CDK molecules are intrinsically linked since CDK activity defines the cell cycle stage of a cell. CDK activity increases through the cell cycle and is responsible for cells progressing through G1, S, G2, and mitosis [44,53] so that an unperturbed asynchronous population of cells in G1 is achieved by a low CDK activity. Thus our results reflect changes happening through the cell cycle as the CDK regulation network undergoes modifications, and an unperturbed cell cycle therefore cannot be uncoupled from CDK activity.".

Overall, the work presented suggests that cell cycle control affects global cellular translation and transcription, which is in line with some studies, but not others. Whilst the study falls short of testing/establishing the (potential) mechanisms involved, these are important findings, which can be used to guide new studies into how the production of biomass is controlled as cells proceed through the cell cycle.

The cell size field, which is considerable and growing, will be interested in this work.

I have expertise in cell cycle control and genome stability, with a focus on the G1-to-S transition and cell cycle checkpoints during interphase.

Reviewer #3 (Evidence, reproducibility and clarity (Required)):

Summary
Basier and Nurse use fission yeast as a model system to investigate how transcription and translation are coupled to cell-cycle progression. They use metabolic labeling in exponentially growing cells and analyze single cells by microscopy. They find that translation scales with size and increases at S/G2 and early mitosis while transcription increases with both size and the amount of DNA. They suggest that changes in CDK activity regulate changes in global translation rates.

Major comments:
1) The paper addresses a much-disputed question in the field. The approach makes the most of the fission-yeast model system and the experiments are beautifully performed. The conclusions are well supported by the data. The experiments are replicated adequately and the statistical analyses are appropriate.

2) The use of cdc25 and in particular the cig1Δ cig2Δ puc1Δ mutants to manipulate cell size is not without challenges when monitoring translation rates. A number of reports in different model organisms suggest that CDK activity can regulate translation. Work from the Nurse lab identified translation factors as CDK substrates (Swaffer et al, 2016), RNApolIII activity and thus tRNA levels are regulated in the cell cycle by CDK in budding yeast (Herrera et al, 2018), phosphorylation of the ribosomal protein RPL12 by CDK1 is required for translation of at least some proteins in mitosis in human cells (Imami et al, 2018), as is phosphorylation of DENR (Clemm von Hohenberg et al, 2022). The authors also suggest that changes in CDK activity might be responsible for the observed changes in global translation rates. It is important to consider whether using mutants impinging on CDK activity might lead to under- or overestimating cell-cycle dependent translation. The authors should either discuss this issue and tune down the hypothesis that CDK activity regulates changes in global translation rates, or use another approach to address the issue. One could use a replication mutant such as cdc17 or cdc20 to alter cell size without interfering with CDK activity. These experiments would strengthen the conclusions and might support the idea that CDK activity regulates changes in

global translation rates.

References

Clemm von Hohenberg K, Müller S, Schleich S, Meister M, Bohlen J, Hofmann TG, Teleman AA (2022) Cyclin B/CDK1 and Cyclin A/CDK2 phosphorylate DENR to promote mitotic protein translation and faithful cell division. Nat Commun 13: 668

Herrera MC, Chymkowitch P, Robertson JM, Eriksson J, Bøe SO, Alseth I, Enserink JM (2018) Cdk1 gates cell cycle-dependent tRNA synthesis by regulating RNA polymerase III activity. Nucleic Acids Res 46: 11698-11711

Imami K, Milek M, Bogdanow B, Yasuda T, Kastelic N, Zauber H, Ishihama Y, Landthaler M, Selbach M (2018) Phosphorylation of the Ribosomal Protein RPL12/uL11 Affects Translation during Mitosis. Mol Cell 72: 84-98 e89

Swaffer MP, Jones AW, Flynn HR, Snijders AP, Nurse P (2016) CDK Substrate Phosphorylation and Ordering the Cell Cycle. Cell 167: 1750-1761 e1716

As discussed above in the reply to reviewer 2, the cell cycle stage and CDK activity are intrinsically linked, CDK activity defines the cell cycle stage so that an increase in CDK activity through the cell cycle is responsible for cells progressing through G1, S, G2, and mitosis (Coudreuse and Nurse, 2010, Swaffer et al., 2016). Therefore, uncoupling CDK activity from the cell cycle is not possible in an unperturbed population. Temperature sensitive mutants of cdc20 (Ramirez et al., 2015, Win et al., 2002) and cdc17 (Jimenez et al., 1992) cause loss of viability when cells are shifted to the restrictive temperature so it cannot be assumed that they are in unperturbed conditions which makes results hard to interpret. It should be noted as far as possible in these experiments we have tried to avoid perturbations. In addition, the fraction of cells permeabilised in our assay decreases significantly when cells are grown above 30 °C, making it difficult to assay such temperature shifts.

Minor comments:

1) The figures are beautifully presented, easy to understand and the cartoons present the experimental strategies very clearly.

2) A major feature of the approach is that translation and transcription are monitored in exponentially growing cells, which are not exposed to any stress such as cell-cycle synchronization. However, one could argue that the analogues used for labeling impose some kind of stress, even if this is not very likely at the labeling times employed. A simple control experiment where the growth rates of labeled and unlabeled cells are compared would strengthen the claim that these are indeed happily growing cells.

It is possible that incubating cells with the analogues could impose some kind of stress on the cell although that could be said about almost any experimental procedure. We have added two supplementary figures with the suggested experiments, showing that incubating cells with EU has little or no impact on their doubling time (we see at most a 2.4 % increase in doubling time in hENT1 hsvTK cells incubated with 20 μM EU, Figure S1I) and that incubating cells with HPG has little impact on their doubling time (we see a 8.6 % increase in doubling time in wild type cells incubated with 10 μM HPG, Figure S1H).

Considering the small impact of analogue incubation on the doubling time of the population, and the fact that cells are only exposed to the analogue for a short time in our assays (compared to continuous growth in the presence of the analogue in the growth curves presented in Figure S1H and I), we conclude that the stress imposed is low.

3) Please comment why the length of the EU labeling differs from figure to figure. In fig 2C, S2C and S2D the labeling on the y axes states 10 min, in Fig 4C it says 20 min.
Please refer to the reply to reviewer 2 on the same topic.

4) Lines 118-119 "The pulse signal was five times the background signal." Figure S2A,B show large variation in signal intensity after 5 min labelling. It is not clear how the pulse signal was estimated to be five times the background signal.
We have added two panels for the supplementary figure 2 showing how the signal to noise ratio was computed for the HPG assay after 5 minutes of incubation (Figure S2G) and for the EU assay after 10 minutes of incubation (Figure S2H).

5) In Fig S4C transcription is up by ca 60 % from G1 to G2, while in Fig 4D transcription is up by ca 25-30%, also from G1 to G2. The only difference I can see is the use of PCNA-GFP. Please comment what the reason might be.
In Figure 4D, transcription is up 33 % from G1 to G2 and in Figure S4C, transcription is up 62 % from the 1C to the 2C population. It is possible that the EGFP-pcn1 strain might have a small growth defect which could possibly explain its lower signal production, the slower growth rate might mean that the concentration of RNA polymerase could be lower in this strain and the dynamic equilibrium model predicts that this would results in a smaller increase from G1 to G2 compared to cells with a higher concentration of RNA polymerase. But obviously this is speculative.

6) Fig 1 B images of unlabeled control cells should also be shown.
We have added 2 panels to the supplementary figure 1 showing the background controls in which cells are fixed immediately after addition of the analogue for the HPG assay (Figure S1F) and for the EU assay (Figure S1G).

7) Lines 156 "to investigate how global cellular translation and transcription are affected by cell size, and by progression through the cell cycle" should be amended. Throughout the description of data in figure 2 binucleated and septated cells were excluded from the analyses, meaning that the data only represent cells in G2. The text should make this clear.
"to investigate how global cellular translation and transcription are affected by cell size, and by progression through the cell cycle" has been changed to "to investigate how global cellular translation and transcription are affected by cell size and by progression through G2" to reflect the fact that binucleated and septated cells are excluded from the analysis on this figure.

8) Lines 241-243 "the S-phase subpopulation was found to have an intermediary global transcription value between the G1 and G2/M subpopulations of around 20-25 %." And Lines 310-313 "the rate of transcription is increased in cells undergoing S-phase by 20 % and is 35 % higher in G2 cells which have completed S-phase, indicating that DNA content is limiting the global rate of transcription." It is unclear what the percentage values refer to and which populations exactly are being compared.
"the S-phase subpopulation was found to have an intermediary global transcription value between the G1 and G2/M subpopulations of around 20-25 %" has been changed to "the S-phase subpopulation was found to have an intermediary global transcription value between the G1 and G2/M subpopulations with an increase of around 20-25 % compared to the G1 subpopulation" and "the rate of transcription is increased in cells undergoing S-phase by 20 % and is 35 % higher in G2 cells which have completed S-phase, indicating that DNA content is limiting the global rate of transcription" has been changed to "the rate of transcription is increased in cells undergoing S-phase by 20 % compared to G1 cells and is 35 % higher in G2 cells which have completed S-phase compared to G1 cells, indicating that DNA content is limiting the global rate of transcription". These changes hopefully will clarify what populations comparisons the percentage values are referring to.

9) Line 85 "Asynchronous cultures ... have not detected" rephrase; change detected to displayed or similar.
"detected" has been changed to "displayed"

10) Line 243 Figure 4J, K should read Figure 4C, D.
"Figure 4j, K" has been changed to "Figure 4D, C"

CROSS-CONSULTATION COMMENTS

I also agree with the comments made by the colleagues. As for the use of the cyclin and cdc25 mutants: I agree with Reviewer #1 that it is unlikely that bulk synthesis rates are conisedarably different, since these strains are going at more or lass normal rates. However, I also agree with reviewer #2 that these mutants cannot be considered as unperturbed conditions. I suspect subtle regulation and in particular cell-cycle dependent regulation might well be lost. At the very least the focus of the interpretation should be on translation/transcription as a function of size, rather than in terms of cell-cycle regulation.

Reviewer #3 (Significance (Required)):

Basier and Nurse address a long-standing question in the cell-cycle field, namely how/whether transcription and translation are coupled to cell-cycle progression. This is technically challenging to address, and many previous studies were hampered by the necessity to synchronize the cells in the cell cycle. The approach of this study of using metabolic labeling in non-synchronized

cells is not novel in itself. However, the analysis by microscopy is superior to previous flow-cytometry based strategies in that it allows the use of cell-cycle markers and thereby precise identification of cells in each cell-cycle phase. In addition, it allows accurate measurements of cell size and thus addressing questions of correlations between cell size and transcription / translation rates. A further strength of the study design is that they investigate both transcription and translation in parallel.
The authors very nicely review the existing literature and point out the likely reasons for conflicting conclusions (synchronization methods, choice of model system). The advantages of their approach, such as single-cell analyses in non-synchronized cells and the use of cell-cycle markers make their conclusions less likely to be flawed and thus represent an important advance in the field.
These findings are of interest for researchers working on the cell-cycle field and on the translation field. There have been significant technical advances in the translation field in recent years, allowing studying not only global translation but also translation of specific mRNAs. I expect that the old questions of coupling cell cycle and cell growth will be revisited also by others, exploiting these new approaches.
My field of expertise extends to the cell-cycle field and the regulation of translation and the use of fission yeast.

Reviewer #4 (Evidence, reproducibility and clarity (Required)):

Summary
Single cell measurements (flow cytometry and imaging) from unperturbed cells are obtained to investigate scaling of transcription and translation in fission yeast. A key finding is that translation and transcription are somewhat differentially responding to changes in cell size and cell cycle. Perhaps the most central finding of this manuscript is that transcription is not a limiting factor to translation and suggests that transcription is not limiting growth (increase in biomass).

Major comments:
What I like in this manuscript is that the translation and transcription measurements have been carefully checked to reflect the initial rates before the HPG and EU signals lose their linearity. More generally, experiments have been conducted with appropriate controls, and the analysis of unperturbed cells in each cell cycle phase is likely to be highly relevant for resolving some of the controversies in the field. Most claims and the conclusions are well supported by the data. Although it is encouraging that the results for translation match the single cell mass measurements in mammalian cells (e.g., ref 18), I would have liked to see some more discussion about the potential caveats of the performed analyses such as the low signal to noise ratio in EU incorporation and other potential technical issues, which might have confounded the results. As an example, looking at Figs 1B and E, most of the protein and RNA synthesis signal is nuclear localized. Is this due to nucleolar staining and incorporation of the labels into nascent ribosomes? Yet the manuscript mentions that roughly half of RNA is for

rRNA and for ribosomal proteins the fraction of HPG incorporation might be even lower. This statement does not sound entirely consistent with the experimental images shown in Fig 1. Please clarify.

We had initially performed modelling to estimate the proportion of rRNA in transcription but after reconsideration we agree that is difficult to assess whether the special pattern we observe is consistent with the statement that roughly half of the nascent RNA is rRNA. There is signal in the cytoplasm indicating that within the pulse time some RNA are exported from the nucleus, thus the localisation of the RNA signal is not necessarily an accurate indication of the fractions of the different RNA types in global transcription. We have removed the statement "Although the precise fractions of the different types of RNA in global transcription have not been fully characterised, recent work indicated that only half of the newly synthesised RNA consists of ribosomal RNA molecules, suggesting that a significant portion of transcription is dedicated to the production of messenger and other RNA molecules [27]." It cannot be concluded that most of the protein synthesis is nuclear located in Figure 1B. As mentioned in the text we cannot differentiate between proteins being synthesised in the nucleus and proteins being rapidly imported, we also cannot say what fraction of the proteins synthesised are related to ribosome biogenesis.

A curious thing that has been glossed over is that the transcription and translation seem not to be completely linear but to display opposite patterns (translation slightly reducing, transcription slightly overshooting with cell size compared to a linear model). It remains possible that this could be experimental noise and a visual pattern that is not real, but it could also be relevant for growth control. For example, my interpretation from Fig. 2B is that the signal is not linear and starts to saturate around 10.5 um cell length as seen from the upper IQR. Related to this, I think it is oversimplification to force the data to appear as a discontinuous linear trend by splitting the data in 2H into two segments. Such a treatment will obviously match the data better than a single linear regression, but perhaps some nonlinear model would be actually much more accurate, unless you can point out some kind of regulatory event at the intersection of these two linear segments. In my opinion the current data looks more like a typical (logarithmic) growth curve of the cell population reaching saturation. Please comment.

We agree that fitting two linear regressions for cells shorter and longer than 15 µm is in Figure 2H and 2I was an oversimplification which could result in a false discontinuity in the data. This echoes a comment from reviewer 1 pointing out that 15 µm might not be the length at which the transition occurs. We have removed the linear regressions and added a locally estimated scatterplot smoothing (LOESS) function which capture the nonlinear transition between the increase of translation with size and the saturation, and we have changed the cell length at the estimated saturation from 18 to 19 µm in the text to better reflect the trend.

The main conclusion presented in the abstract is that scaling in transcription may result from dynamic equilibrium between RNA polymerases and available DNA template. This is a bit of speculative part, which I was not too fond of. The dynamic equilibrium idea has been suggested

Review COMMONS

also elsewhere (refs 47) and is not well developed in this manuscript. There is a lack of mechanistic understanding and no formal (mathematical) model to support this idea. For example, global transcription increases much less (1.3-1.4x) than expected based on the increase in DNA content from G1 to G2 (2x). Is this expected based on the dynamic equilibrium model?

The dynamic equilibrium model has been proposed and developed by Swaffer et al. (2022 – currently on bioRxiv) based on mass action kinetics describing the interaction between RNA polymerases and DNA. The model predicts that transcription increases with cell size and with the amount of DNA. With this model, the increase in transcription with DNA for a given cell size is also a function of cell size. Smaller cells are predicted to have a smaller relative increase in transcription from 1C to 2C DNA content than larger cells. This implies that depending on the cell size to DNA ratio of a cell, the span increase in transcription produced by a doubling in the amount of DNA goes from a small increase (at small cell size to DNA ratio) to a doubling (at large cell size to DNA ratio). Thus, in our view the 1.3-1.4x increase in transcription we observe from G1 to G2 is consistent with the dynamic equilibrium model.

I am somewhat concerned about the interpretation of the S phase data in the global transcription measurement. The quantification in Fig. 4D shows S phase being intermediate between G1 and G2. Yet, when you look at the data in Fig. 4C, the S phase median is clearly discontinuous, with higher transcription in smaller S cells. I believe this could affect the normalized data in Fig. 4D and result in the apparent increase in transcription in S phase cells. Having said that, I am not sure if this small S phase transcription is noise (low cell counts?) or a real S phase specific regulation of transcription which is not DNA content dependent. This results is one of the most central ones in this paper to differentiate between transcriptional and translational scaling. Therefore, additional data or insights would be highly appreciated.

It is possible that the discontinuity in the medians of the S phase population in Figure 4C could be the result of noise due to the low cell count in the short size bins (115 cells at 6.5 µm, 404 at 7.5 µm). In addition, because we cannot measure DNA with a degree of accuracy high enough to identify how advanced in S-phase each cell is, we do not know the distribution of the advancement into S-phase of cells for each length bin. This is complicated by the fact that some cells of the CCP∆ mutant start S-phase whilst still septated and might be in a late S-phase stage by the time the cell splits so the median global transcription of the shorter length bin does not necessary reflect the median of early S-phase cells. Hence the discontinuity observed with cell length does not necessarily suggest that there is a discontinuity happening through S-phase. We suggest that since the mean global transcription per cell length of cells in S-phase is in between the mean global transcription per cell length of cells in G1 and in G2, the increase happens through S-phase. To reflect this possibility we have added "It is also possible that the increase happens at a certain stage of S-phase independent of the amount of DNA since we do not know the extent of S-phase of each cell."

Minor comments:
Line 61: "patterns of protein RNA". I guess this refers to patterns of protein/RNA synthesis?
"patterns of protein and RNA" has been changed to "patterns of protein and RNA synthesis".
Line 248: typo "Tanslation"
"Tanslation" has been changed to "Translation".
Line 410 and 416: Move interquartile ranges from line 416 to line 410 as this is the first occurrence of the IQR abbreviation.
"Interquartile ranges" has been moved from line 416 to line 410.
Line 473: "Almost linear". This is a subjective expression, please provide some measure such as the R2 value to quantitatively evaluate linearity in this strain.
We have added a measure of the deviation from linearity in the text ", 15 % deviation from the OLS linear regression shown in Figure 1F".
Line 547: Is there a reason to stress in this experiment that the AREA of the fluorescence signal was measured as the area indicates the total fluorescence intensity?
"area of the" has been replaced by "total" so the sentence refers to the total fluorescence intensity signal of Sytox Green.
Fig1A: The schematic mentions peptides, shouldn't it be more accurate to use "polypeptides" or "proteins" when discussing protein synthesis?
"***Peptides has been changed to Polypeptides"***
Fig 5G: Y axis scale has a typo in the word transcription.
"Trancription" has been changed to "Transcription"

CROSS-CONSULTATION COMMENTS
I also agree with the points raised by the colleagues. There will always be some technical or interpretation issues related to every experimental technique, every model system and every mutant strain used. I believe after addressing these limitations as pointed out in the reviews, most of those issues have been clarified for the readers.

Reviewer #4 (Significance (Required)):

Basier and Nurse revisit the classic question regarding growth and cell size control by examining scaling of global translation and transcription in fission yeast.
Knowing how cells alter their transcription and translation has important consequences in cellular functions during proliferation and cellular aging and is of broad general interest. The main driver for this current work is that previous experiments both in fission yeast and other model organisms have yielded conflicting results, possibly due to different cell cycle synchronization methods. The strength of the paper is indeed in the single cell analyses of well defined yeast strains which allow accurate assessment of the cell cycle dependent changes and accurate measurements of cell size using the cell length.

Reassuringly, the single cell analyses from unperturbed yeast cells resemble those recently obtained from unperturbed growth of individual mammalian cells. The main conclusion that

transcription is not limiting translation, and consequently not limiting growth of the cells, is interesting as it is not consistent with some of the prevailing ideas in the cell size field. These ideas include ploidy dependent gene expression where DNA content is thought to be limiting growth or the model for minimal gene expression which assumes RNA polymerases are limiting gene expression and growth. In this regard, this manuscript provides important insights for future thinking of how growth is controlled.

keywords: cell cycle, cell size control

1st Editorial Decision 6th Feb 2023

Thank you for submitting your revised Review Commons manuscript for consideration by The EMBO Journal. In light of the constructive original comments and the overall interest of the topic, I decided to treat the study similar to a regular revision, and sent it directly to the four original referees for an assessment of your responses. Despite noting that the underlying mechanisms remain to be fully elucidated, all referees acknowledge the revision work and are in principle supportive of eventual publication, following incorporation of some additional minor modifications. As you will see from the comments below, referees 1 and 4 still retain a few specific concerns (partly arising from the revision experiments), which I would invite you to address during a final round of revision; following which we would be happy to offer publication of this work in The EMBO Journal. Should you have any questions/comments regarding these queries and how to best address them, please do not hesitate to contact me, and I would be happy to discuss them directly with you.

In addition to the scientific revisions, please at this stage also adjust the manuscript according to our formatting guidelines and incorporate the following editorial points:

Referee #1:

Basier and Nurse address all of my comments, but in so doing they raise another point that could be addressed before publication. In Figures 3B, 3H, 4B and S4A, they categorize cells as G1 or G2/M (or 1C or 2C, they should be consistent in their nomenclature), using DNA-staining intensity and length. However, in Figures 3H and 4B, they are actually able to categorize the cells into G1, S and G2/M, with the addition of PCNA data, as demonstrated in Figures 3I, 3K, 4D and S4D. It would, therefore, be informative to show all three categories in Figures 3H and 4B, instead of mixing the S-phase cells in with the G1 and G2?M cells.

Referee #2:

Knowing how cells alter their transcription and translation has important consequences in cellular functions during proliferation and cellular aging and is of broad general interest.
The experiments have been carefully conducted and the conclusions drawn are supported by the data.
I reviewed this manuscript previously and I am satisfied with the author responses and the modifications in the manuscript.

Referee #3:

Overall, the work presented suggests that cell cycle control affects global cellular translation and transcription, which is in line with some studies, but not others. The assays and data analysis are robust, and the data supports the general conclusions. The authors have addressed all my concerns adequately in their rebuttal and revised manuscript.

These are important findings, which can be used to guide new studies into how the production of biomass is controlled as cells proceed through the cell cycle. This will be of great interest to the cell size field, which is considerable and growing, but the study falls short of testing/establishing the (potential) mechanisms involved which takes away from its potential impact.

Referee #4:

Summary:
Basier and Nurse use fission yeast as a model system to investigate how transcription and translation are coupled to cell-cycle progression. They use metabolic labeling in exponentially growing cells and analyze single cells by microscopy. They find that translation scales with size and increases at S/G2 and early mitosis while transcription increases with both size and the amount of DNA. They suggest that changes in CDK activity regulate changes in global translation rates.
The revised version has been improved on all the minor issues and they fully exploit the advantages the fission yeast model has to offer. The data analyses are robust and the conclusions are mainly supported (but see below).

Major comment
However, the added discussion emphasizes the idea that the cell-cycle dependent changes in translation rates are due to CDK activity even more than the original manuscript ("our results reflect changes happening through the cell cycle as the CDK regulation network undergoes modifications"). This is an attractive hypothesis but not proven. In fact, the 35-40% increase in translation in the G2 cells compared with G1 cells of similar size (Figure 3C, D) in the CCPΔ mutant argues against CDK regulation being responsible, even if CDK-regulating pathways other than cyclins are in place.
Using different media as reviewer 2 suggested would result in longer G1 without impairing CDK regulation. I appreciate the argument that this would most likely be achieved through regulating CDK activity, but in this situation any and all CDK regulation

would at least be intact. It would be particularly interesting to also use nutritional conditions which increase the generation time without prolonging G1 (Cys or Gly) (PMID: 10036243), although little is known about cell-cycle regulation under these conditions and thus this line of experiments might go beyond the scope of the paper.
Alternatively, the caveats associated with the use of the CCPΔ mutant should be highlighted and the hypothesis attributing the regulation of translation rates to CDK clearly presented as a hypothesis.
Furthermore, the authors state themselves that "unperturbed cell cycle therefore cannot be uncoupled from CDK activity". Therefore, a strain carrying mutations affecting CDK activity can hardly be described as "unperturbed".

Minor point
I found the comparison to the conclusions of the Stonyte et al paper in the response to Reviewer 2 interesting and useful. I'd suggest including a shorter version in the paper, to highlight the differences in the two studies using unperturbed conditions and single-cell approaches.

Referee #1:

Basier and Nurse address all of my comments, but in so doing they raise another point that could be addressed before publication. In Figures 3B, 3H, 4B and S4A, they categorize cells as G1 or G2/M (or 1C or 2C, they should be consistent in their nomenclature), using DNA-staining intensity and length. However, in Figures 3H and 4B, they are actually able to categorize the cells into G1, S and G2/M, with the addition of PCNA data, as demonstrated in Figures 3I, 3K, 4D and S4D. It would, therefore, be informative to show all three categories in Figures 3H and 4B, instead of mixing the S-phase cells in with the G1 and G2?M cells.

Figure 3H and 4B did not show cells categorised as S-phase, so we have added panels to Figure EV3 and Figure EV4 to show where the cells in S-phase would be on Figure 3H (Figure EV3E) and Figure 4B (Figure EV4E). We initially categorised cells based solely on their DNA intensity which is why we used the 1C/2C DNA content nomenclature (1C cells could be in G1 or early S-phase, and 2C cells could be in late S-phase, G2, or M). The addition of the PCNA marker allows for a more accurate categorisation of cells in S-phase and we are confident that using this technique the cells can be categorised as in G1, S, or G2/M so we think the G1 and G2/M nomenclature is more appropriate than the 1C/2C for these cells.

Referee #2:

Knowing how cells alter their transcription and translation has important consequences in cellular functions during proliferation and cellular aging and is of broad general interest.
The experiments have been carefully conducted and the conclusions drawn are supported by the data.
I reviewed this manuscript previously and I am satisfied with the author responses and the modifications in the manuscript.

Referee #3:

Overall, the work presented suggests that cell cycle control affects global cellular translation and transcription, which is in line with some studies, but not others. The assays and data analysis are robust, and the data supports the general conclusions. The authors have addressed all my concerns adequately in their rebuttal and revised manuscript.

These are important findings, which can be used to guide new studies into how the production of biomass is controlled as cells proceed through the cell cycle. This will be of great interest to the cell size field, which is considerable and growing, but the study falls short of testing/establishing the (potential) mechanisms involved which takes away from its potential impact.

Referee #4:

Summary:
Basier and Nurse use fission yeast as a model system to investigate how transcription and translation are coupled to cell-cycle progression. They use metabolic labeling in exponentially growing cells and analyze single cells by microscopy. They find that translation scales with size and increases at S/G2 and early mitosis while transcription increases with both size and the amount of DNA. They suggest that changes in CDK activity regulate changes in global translation rates.
The revised version has been improved on all the minor issues and they fully exploit the advantages the fission yeast model has to offer. The data analyses are robust and the conclusions are mainly supported (but see below).

Major comment
However, the added discussion emphasizes the idea that the cell-cycle dependent changes in translation rates are due to CDK activity even more than the original manuscript ("our results reflect changes happening through the cell cycle as the CDK regulation network undergoes modifications"). This is an attractive hypothesis but not proven. In fact, the 35-40% increase in translation in the G2 cells compared with G1 cells of similar size (Figure 3C, D) in the CCPΔ mutant argues against CDK regulation being responsible, even if CDK-regulating pathways other than cyclins are in place.
Using different media as reviewer 2 suggested would result in longer G1 without impairing CDK regulation. I appreciate the argument that this would most likely be achieved through regulating CDK activity, but in this situation any and all CDK regulation would at least be intact. It would be particularly interesting to also use nutritional conditions which increase the generation time without prolonging G1 (Cys or Gly) (PMID: 10036243), although little is known about cell-cycle regulation under these conditions and thus this line of experiments might go beyond the scope of the paper.
Alternatively, the caveats associated with the use of the CCPΔ mutant should be highlighted and the hypothesis attributing the regulation of translation rates to CDK clearly presented as a hypothesis.
Furthermore, the authors state themselves that "unperturbed cell cycle therefore cannot be uncoupled from CDK activity". Therefore, a strain carrying mutations affecting CDK activity can hardly be described as "unperturbed".

We have added *"It is possible however, that in the absence of the cyclins Cig1, Cig2, and Puc1, cell cycle transitions, particularly the G1/S transition could have some differences compared with the wild type."* (line 372) This reflects possible changes due to the *CCPΔ* mutant.

We have changed "*Perhaps changes in CDK activity through the cell cycle could influence the fraction of active ribosomes and be responsible for the cell cycle related changes in translation*" to "*We hypothesise that changes in CDK activity through the cell cycle could influence the fraction of active ribosomes and be responsible for the cell cycle related changes in translation*" (line 386) to clearly show that attributions of the regulation of translation rates to CDK is an hypothesis.
We have changed "*an unperturbed cell cycle cannot therefore be uncoupled from CDK activity*" to "*in steady state cells the cell cycle cannot be uncoupled from CDK activity*" (line 371) to reflect that the *CCPΔ* mutation could be seen as a perturbation of the wild type cell cycle but that in a population growing and dividing at steady state it is not possible to perturb CDK activity without perturbing the cell cycle and thus no longer being in the steady state.

Minor point
I found the comparison to the conclusions of the Stonyte et al paper in the response to Reviewer 2 interesting and useful. I'd suggest including a shorter version in the paper, to highlight the differences in the two studies using unperturbed conditions and single-cell approaches.

We have added "*and with work in asynchronous cultures of fission yeast grown in minimal medium containing isoleucine as a nitrogen source which showed no increase from G1 to S and an increase from S to G2 (Stonyte et al, 2018). In addition, our assay allows the identification of different stages of mitosis which was not previously possible in Stonyte et al, and shows that global translation follows the same increase and decrease patterns observed in mammalian cells (Miettinen et al, 2019).*" (line 322) to include the discussion about the *Stonyte et al.* work.

In addition to the scientific revisions, please at this stage also adjust the manuscript according to our formatting guidelines and incorporate the following editorial points:

- Please download and complete our author checklist (link provided below).
Done

- Please upload the manuscript text as a separate DOCX file, and all main Figures (as well as any Expanded View figures) as individual files with sufficient resolution/quality for production.

Done

- "Supplementary information" at EMBO Press has been replaced by Expanded View and Appendix (see embopress.org/page/journal/14602075/authorguide#expandedview for further guidance). Therefore, I would suggest to convert Figures S1-S4 into "Expanded View figures" (to be referenced in text and legends as "Figure EV1/2/...") - they will in this case be type-set and directly viewable in the HTML version of the paper.

Supplementary information has been replaced by Expanded View figures and Figures S1-S4 have been renamed Figures EV1-4 accordingly

- Please adjust the format of the reference list and of the in-text citations according to EMBO Journal format (alphabetical order, author name et al + year...).

Done

- Please include a dedicated "Data Availability" section at the end of the Material and Methods (see embopress.org/page/journal/14602075/authorguide#dataavailability for its format); should there no data deposition to public repositories linked to the study, this should still be stated as "This study includes no data deposited in external repositories."

- Please adjust the header and the format of the Disclosure and competing interests statement (next to the Acknowledgment section) as specified in our Guide to Authors - for details, see https://www.embopress.org/competing-interests

Done

- As we are switching from a free-text author contribution statement towards a more formal statement based on Contributor Role Taxonomy (CRediT) terms, please specify each author's contribution(s) directly in the Author Information page of our submission system during upload of the final manuscript, and remove the current Author Contribution section in the text. See https://casrai.org/credit/ for more information.

Done

- I am wondering whether the title could be made somewhat more informative and broadly appealing by including some information about key findings/conclusions of the study, e.g. regarding cell cycle control affecting global cellular translation and transcription?

Proposition for new:
The cell cycle and cell size influence the rates of global cellular translation and transcription in fission yeast.

- Finally, please provide suggestions for a short 'blurb' text prefacing and summing up the conceptual aspect of the study in two sentences (max. 250 characters), followed by 3-5 one-sentence 'bullet points' with brief factual statements of key results of the paper; they will form the basis of an editor-written 'Synopsis' accompanying the online version of the article. Please also upload a synopsis image, which can be used as a "visual title" for the synopsis section of your paper. The image should be in PNG or JPG format, and please

make sure that it remains in the modest dimensions of (exactly) 550 pixels wide and 300-600 pixels high.

Proposition for short “blurb”:

We developed an assay using exponentially growing populations of fission yeast to understand the influences of the cell cycle and cell size on the global rates of production of proteins and RNA in unperturbed cells.

-Both global transcription and translation increase with cell size.

-In large cells, global translation becomes limited by cell size before transcription does.

-Global translation is regulated by cell cycle events, increasing at the S/G2 transition and early in mitosis, and deceasing later in mitosis.

-Global transcription seem to increase through S phase.

Thank you for submitting your re-revised manuscript for our consideration. It has now been checked by myself as well as by our data editors, and I am happy to say is now ready to be accepted in principle. Before being able to send a formal letter of acceptance, there are only the following editorial points that still need to be addressed:

EMBO Press Author Checklist

Corresponding Author Name: Clovis Basier	
Journal Submitted to: The EMBO Journal	
Manuscript Number: EMBOJ-2022-113333	

USEFUL LINKS FOR COMPLETING THIS FORM
The EMBO Journal - Author Guidelines
EMBO Reports - Author Guidelines
Molecular Systems Biology - Author Guidelines
EMBO Molecular Medicine - Author Guidelines

Reporting Checklist for Life Science Articles (updated January
This checklist is adapted from Materials Design Analysis Reporting (MDAR) Checklist for Authors. MDAR establishes a minimum set of requirements in transparent reporting in the life sciences (see Statement of Task: 10.31222/osf.io/9sm4x). Please follow the journal's guidelines in preparing your
Please note that a copy of this checklist will be published alongside your article.

Abridged guidelines for figures
1. Data
The data shown in figures should satisfy the following conditions:
- the data were obtained and processed according to the field's best practice and are presented to reflect the results of the experiments in an accurate and unbiased manner.
- ideally, figure panels should include only measurements that are directly comparable to each other and obtained with the same assay.
- plots include clearly labeled error bars for independent experiments and sample sizes. Unless justified, error bars should not be shown for technical
- if n<5, the individual data points from each experiment should be plotted. Any statistical test employed should be justified.
- Source Data should be included to report the data underlying figures according to the guidelines set out in the authorship guidelines on Data

2. Captions
Each figure caption should contain the following information, for each panel where they are relevant:
- a specification of the experimental system investigated (eg cell line, species name).
- the assay(s) and method(s) used to carry out the reported observations and measurements.
- an explicit mention of the biological and chemical entity(ies) that are being measured.
- an explicit mention of the biological and chemical entity(ies) that are altered/varied/perturbed in a controlled manner.
- the exact sample size (n) for each experimental group/condition, given as a number, not a range;
- a description of the sample collection allowing the reader to understand whether the samples represent technical or biological replicates (including how many animals, litters, cultures, etc.).
- a statement of how many times the experiment shown was independently replicated in the laboratory.
- definitions of statistical methods and measures:
  - common tests, such as t-test (please specify whether paired vs. unpaired), simple χ2 tests, Wilcoxon and Mann-Whitney tests, can be unambiguously identified by name only, but more complex techniques should be described in the methods section;
  - are tests one-sided or two-sided?
  - are there adjustments for multiple comparisons?
  - exact statistical test results, e.g., P values = x but not P values < x;
  - definition of 'center values' as median or average;
  - definition of error bars as s.d. or s.e.m.

Please complete ALL of the questions below.
Select "Not Applicable" only when the requested information is not relevant for your study.

Materials

Newly Created Materials	Information included in the manuscript?	In which section is the information available? (Reagents and Tools Table, Materials and Methods, Figures, Data Availability Section)
New materials and reagents need to be available; do any restrictions apply?	Not Applicable	

Antibodies	Information included in the manuscript?	In which section is the information available? (Reagents and Tools Table, Materials and Methods, Figures, Data Availability Section)
For **antibodies** provide the following information:<br>- Commercial antibodies: RRID (if possible) or supplier name, catalogue number and or/clone number<br>- Non-commercial: RRID or citation	Not Applicable	

DNA and RNA sequences	Information included in the manuscript?	In which section is the information available? (Reagents and Tools Table, Materials and Methods, Figures, Data Availability Section)
**Short novel DNA or RNA including primers, probes**: provide the sequences.	Yes	Materials and Methods

Cell materials	Information included in the manuscript?	In which section is the information available? (Reagents and Tools Table, Materials and Methods, Figures, Data Availability Section)
**Cell lines:** Provide species information, strain. Provide accession number in repository **OR** supplier name, catalog number, clone number, and/**OR** RRID.	Not Applicable	
**Primary cultures:** Provide species, strain, sex of origin, genetic modification status.	Not Applicable	
Report if the cell lines were recently **authenticated** (e.g., by STR profiling) and tested for mycoplasma contamination.	Not Applicable	

Experimental animals	Information included in the manuscript?	In which section is the information available? (Reagents and Tools Table, Materials and Methods, Figures, Data Availability Section)
**Laboratory animals or Model organisms:** Provide species, strain, sex, age, genetic modification status. Provide accession number in repository **OR** supplier name, catalog number, clone number, **OR** RRID.	Not Applicable	
**Animal observed in or captured from the field:** Provide species, sex, and age where possible.	Not Applicable	
Please detail **housing and husbandry conditions**.	Not Applicable	

Plants and microbes	Information included in the manuscript?	In which section is the information available? (Reagents and Tools Table, Materials and Methods, Figures, Data Availability Section)
**Plants:** provide species and strain, ecotype and cultivar where relevant, unique accession number if available, and source (including location for collected wild specimens).	Not Applicable	
**Microbes:** provide species and strain, unique accession number if available, and source.	Yes	Materials and Methods

Human research participants	Information included in the manuscript?	In which section is the information available? (Reagents and Tools Table, Materials and Methods, Figures, Data Availability Section)
If collected and within the bounds of privacy constraints report on age, sex and gender or ethnicity for all study participants.	Not Applicable	

Core facilities	Information included in the manuscript?	In which section is the information available? (Reagents and Tools Table, Materials and Methods, Figures, Data Availability Section)
If your work benefited from core facilities, was their service mentioned in the acknowledgments section?	Yes	Acknowledgements

Design

Study protocol	Information included in the manuscript?	In which section is the information available? (Reagents and Tools Table, Materials and Methods, Figures, Data Availability Section)
If study protocol has been **pre-registered, provide DOI in the manuscript**. For clinical trials, provide the trial registration number **OR** cite DOI.	Not Applicable	
Report the **clinical trial registration number** (at ClinicalTrials.gov or equivalent), where applicable.	Not Applicable	

Laboratory protocol	Information included in the manuscript?	In which section is the information available? (Reagents and Tools Table, Materials and Methods, Figures, Data Availability Section)
Provide DOI OR other citation details if **external detailed step-by-step protocols** are available.	Not Applicable	

Experimental study design and statistics	Information included in the manuscript?	In which section is the information available? (Reagents and Tools Table, Materials and Methods, Figures, Data Availability Section)
Include a statement about **sample size** estimate even if no statistical methods were used.	Yes	Figures
Were any steps taken to minimize the effects of subjective bias when allocating animals/samples to treatment (e.g. **randomization procedure**)? If yes, have they been described?	Not Applicable	
Include a statement about **blinding** even if no blinding was done.	Not Applicable	
Describe **inclusion/exclusion criteria** if samples or animals were excluded from the analysis. Were the criteria pre-established?<br><br>If sample or data points were omitted from analysis, report if this was due to attrition or intentional exclusion and provide justification.	Not Applicable	
For every figure, are **statistical tests** justified as appropriate? Do the data meet the assumptions of the tests (e.g., normal distribution)? Describe any methods used to assess it. Is there an estimate of variation within each group of data? Is the variance similar between the groups that are being statistically compared?	Yes	Figures

Sample definition and in-laboratory replication	Information included in the manuscript?	In which section is the information available? (Reagents and Tools Table, Materials and Methods, Figures, Data Availability Section)
In the figure legends: state number of times the experiment was **replicated** in laboratory.	Yes	Figures
In the figure legends: define whether data describe **technical or biological replicates**.	Yes	Figures

Ethics

Ethics	Information included in the manuscript?	In which section is the information available? (Reagents and Tools Table, Materials and Methods, Figures, Data Availability Section)
Studies involving **human participants**: State details of **authority granting ethics approval** (IRB or equivalent committee(s), provide reference number for approval.	Not Applicable	
Studies involving **human participants**: Include a statement confirming that **informed consent** was obtained from all subjects and that the experiments conformed to the principles set out in the WMA Declaration of Helsinki and the Department of Health and Human Services Belmont Report.	Not Applicable	
Studies involving **human participants:** For publication of **patient photos**, include a statement confirming that consent to publish was obtained.	Not Applicable	
Studies involving experimental **animals**: State details of **authority granting ethics approval** (IRB or equivalent committee(s), provide reference number for approval. Include a statement of compliance with ethical regulations.	Not Applicable	
Studies involving **specimen and field samples:** State if relevant **permits** obtained, provide details of authority approving study; if none were required, explain why.	Not Applicable	

Dual Use Research of Concern (DURC)	Information included in the manuscript?	In which section is the information available? (Reagents and Tools Table, Materials and Methods, Figures, Data Availability Section)
Could your study fall under dual use research restrictions? Please check biosecurity documents and list of **select agents and toxins** (CDC): https://www.selectagents.gov/sat/list.htm	Not Applicable	
If you used a select agent, is the security level of the lab appropriate and reported in the manuscript?	Not Applicable	
If a study is subject to dual use research of concern regulations, is the name of the **authority granting approval and reference number** for the regulatory approval provided in the manuscript?	Not Applicable	

Reporting

The MDAR framework recommends adoption of discipline-specific guidelines, established and endorsed through community initiatives. Journals have their own policy about requiring specific guidelines and recommendations to complement MDAR.

Adherence to community standards	Information included in the manuscript?	In which section is the information available? (Reagents and Tools Table, Materials and Methods, Figures, Data Availability Section)
State if relevant guidelines or checklists (e.g., **ICMJE, MIBBI, ARRIVE, PRISMA**) have been followed or provided.	Not Applicable	
For **tumor marker prognostic studies,** we recommend that you follow the **REMARK** reporting guidelines (see link list at top right). See author guidelines, under 'Reporting Guidelines'. Please confirm you have followed these guidelines.	Not Applicable	
For **phase II and III randomized controlled trials**, please refer to the **CONSORT** flow diagram (see link list at top right) and submit the CONSORT checklist (see link list at top right) with your submission. See author guidelines, under 'Reporting Guidelines'. Please confirm you have submitted this list.	Not Applicable	

Data Availability

Data availability	Information included in the manuscript?	In which section is the information available? (Reagents and Tools Table, Materials and Methods, Figures, Data Availability Section)
Have **primary datasets** been deposited according to the journal's guidelines (see 'Data Deposition' section) and the respective accession numbers provided in the Data Availability Section?	Not Applicable	
Were **human clinical and genomic datasets** deposited in a public access-controlled repository in accordance to ethical obligations to the patients and to the applicable consent agreement?	Not Applicable	
Are **computational models** that are central and integral to a study available without restrictions in a machine-readable form? Were the relevant accession numbers or links provided?	Not Applicable	
If publicly available data were reused, provide the respective **data citations in the reference list**.	Not Applicable	
